# Cryptic frenulates are the dominant chemosymbiotrophic fauna at Arctic and high latitude Atlantic cold seeps

**DOI:** 10.1371/journal.pone.0209273

**Published:** 2018-12-28

**Authors:** Arunima Sen, Sébastien Duperron, Stéphane Hourdez, Bérénice Piquet, Nelly Léger, Andrey Gebruk, Anne-Sophie Le Port, Mette Marianne Svenning, Ann C. Andersen

**Affiliations:** 1 Centre for Arctic Gas Hydrate, Environment and Climate (CAGE), UiT The Arctic University of Norway, Tromsø, Norway; 2 Sorbonne Université, UMR7208 (MNHN, CNRS, IRD, UCN, UA) Biologie des organismes et écosystèmes aquatiques (BOREA), Paris, France; 3 Muséum National d’Histoire Naturelle–UMR7245 (MNHN CNRS) Mécanismes de Communication et Adaptation des Micro-organismes (MCAM), Paris, France; 4 UMR7144 Sorbonne Université, CNRS–Equipe Adaptation et Biologie des Invertébrés Marins en Conditions Extrêmes (ABICE)—Station Biologique de Roscoff, Roscoff, France; 5 Shirshov Institute of Oceanology, Moscow, Russia; 6 Department of Arctic Marine Biology, UiT The Arctic University of Norway, Tromsø, Norway; Naturhistoriska riksmuseet, SWEDEN

## Abstract

We provide the first detailed identification of Barents Sea cold seep frenulate hosts and their symbionts. Mitochondrial COI sequence analysis, in combination with detailed morphological investigations through both light and electron microscopy was used for identifying frenulate hosts, and comparing them to *Oligobrachia haakonmosbiensis* and *Oligobrachia webbi*, two morphologically similar species known from the Norwegian Sea. Specimens from sites previously assumed to host *O*. *haakonmosbiensis* were included in our molecular analysis, which allowed us to provide new insight on the debate regarding species identity of these *Oligobrachia* worms. Our results indicate that high Arctic seeps are inhabited by a species that though closely related to *Oligobrachia haakonmosbiensis*, is nonetheless distinct. We refer to this group as the *Oligobrachia* sp. CPL-clade, based on the colloquial names of the sites they are currently known to inhabit. Since members of the *Oligobrachia* sp. CPL-clade cannot be distinguished from *O*. *haakonmosbiensis* or *O*. *webbi* based on morphology, we suggest that a complex of cryptic *Oligobrachia* species inhabit seeps in the Norwegian Sea and the Arctic. The symbionts of the *Oligobrachia* sp. CPL-clade were also found to be closely related to *O*. *haakonmosbiensis* symbionts, but genetically distinct. Fluorescent *in situ* hybridization and transmission electron micrographs revealed extremely dense populations of bacteria within the trophosome of members of the *Oligobrachia* sp. CPL-clade, which is unusual for frenulates. Bacterial genes for sulfur oxidation were detected and small rod shaped bacteria (round in cross section), typical of siboglinid-associated sulfur-oxidizing bacteria, were seen on electron micrographs of trophosome bacteriocytes, suggesting that sulfide constitutes the main energy source. We hypothesize that specific, local geochemical conditions, in particular, high sulfide fluxes and concentrations could account for the unusually high symbiont densities in members of the *Oligrobrachia* sp. CPL-clade.

## 1. Introduction

The annelid clade Siboglinidae consists of tube dwelling worms that obtain nutrition from endosymbiotic bacteria, usually fixing carbon through the oxidation of reduced compounds [1–3]. As a result, this group tends to inhabit reducing environments and due to their prominence at hydrothermal (‘hot’) vents and hydrocarbon (‘cold’) seeps, they have received considerable attention. However, this attention is skewed towards the large-bodied and highly publicized vestimentiferan lineage. The most speciose group within Siboglinidae is the frenulate lineage [1,3,4], which is more basal within the clade [5,6] and potentially representative of the most common symbioses in the deep sea [7]. Despite the abundance of frenulate species and their wide distribution, for a long time, only five frenulate species were included in molecular phylogenetic analyses [4,8]. Recently, more frenulate species have been studied from both a molecular and morphological perspective [9–13], but due to an overall paucity of information, when DNA analyses have been carried out, molecular operational taxonomic units could not always be assigned to nominal species [10]. It is now well established that one of the reasons why major relationships within siboglinids is poorly understood is an inadequate sampling of frenulates [3,4]. In high latitude regions of the Atlantic Ocean, such as in the Norwegian Sea, frenulates and moniliferan siboglinids constitute the dominant chemosymbiotrophic megafauna at sites of hydrocarbon seepage (cold seeps) [14–17]. Further north, in the high Arctic, this scenario changes such that frenulates are the sole confirmed chemosymbiotrophic megafauna of cold seeps [18–21]. This means that at seeps in the high Arctic, frenulates alone are likely responsible for ecosystem engineering roles (altering sediment geochemistry, driving trophic pathways and energy transfer, determining or creating niches and habitats, etc.) [22–27] that are usually executed collectively by multiple dominant chemosynthesis based megafaunal species. Therefore, identifying resident frenulate species and characterizing their symbionts is critical for understanding the poorly studied ecosystems of Arctic cold seeps. To date, the frenulates of just two Arctic seeps have been identified: two stations of a shallow seep site in the Laptev Sea and a seep north-east of Svalbard. Both of these localities have been characterized as hosting the frenulate, *Oligobrachia haakonmosbiensis* [13,20], which was originally described from the Håkon Mosby mud volcano (HMMV) [12]. However, these identifications were based only on morphology, though DNA sequences for *O*. *haakonmosbiensis* are available [11]. A complicating factor is the question of whether *O*. *haakonmosbiensis* ought to even be considered a distinct species, since it is morphologically extremely similar to *Oligobrachia webbi*, a frenulate described from off the coast of Tromsø, northern Norway [28]. A taxonomic revision upheld the species status of *O*. *haakonmosbiensis* and extended the distribution of the species to a number of sites in the Norwegian Sea and the Arctic Ocean [13]. Once again though, morphology was the only basis for species identification and no molecular data was obtained or used to compare the samples, which span large geographic distances. Subsequently, frenulates from other Arctic seep sites have been either identified as being similar to *O*. *haakonmosbiensis* [18], or the issue of species identity has been left open ended [19,21].

**Fig 1:**
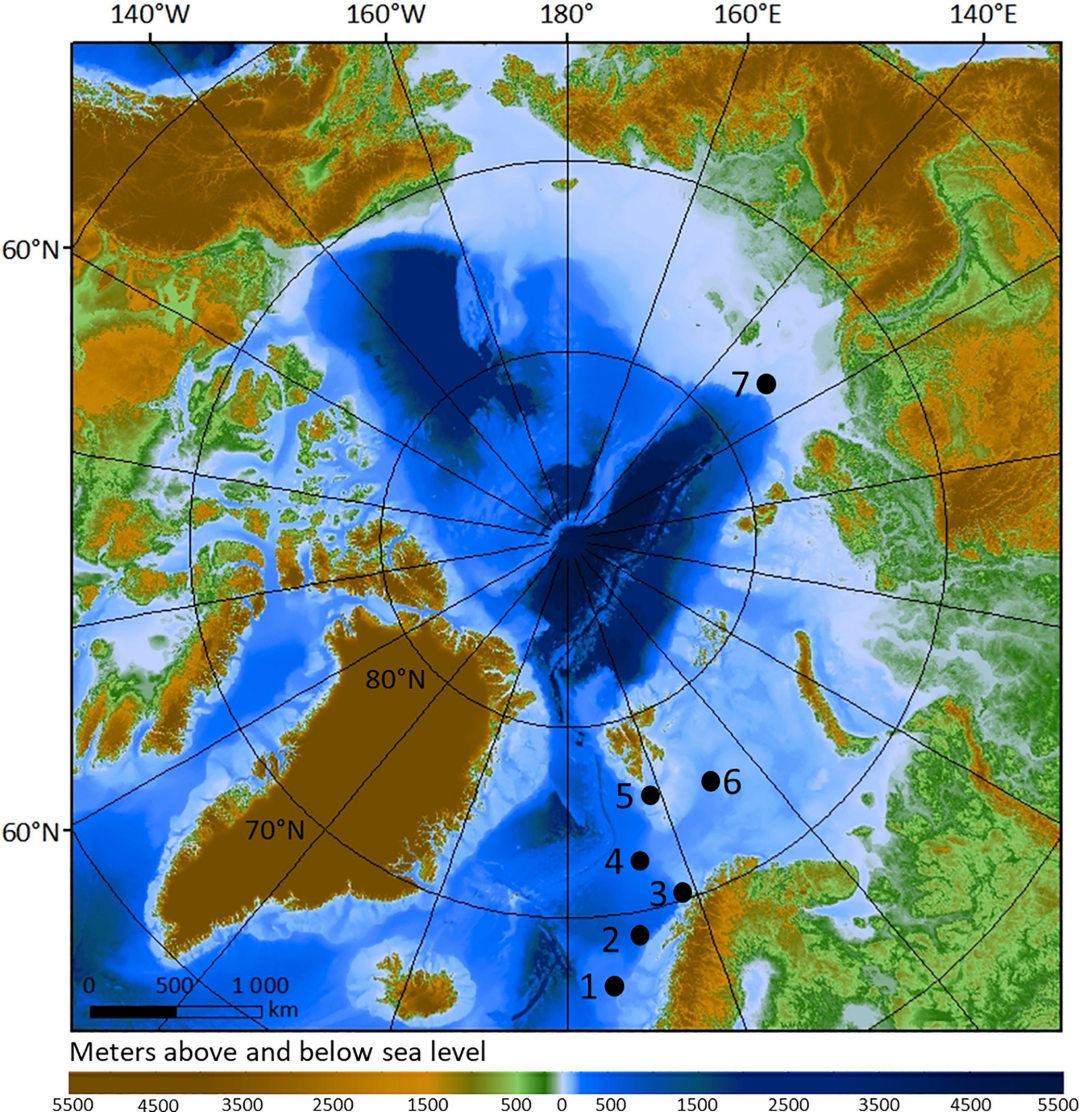
Map of sample locations. Composite map of the sampling sites used in this study, as well as the type locality of *O*. *webbi*. 1: Nyegga/Storegga slide, 2: Lofoten canyon site, 3: type locality of *O*. *webbi*, off Kvaløya, Tromsø, 4: Håkon Mosby mud volcano (type locality of *O*. *haakonmosbiensis*, 5: pingo site, 6: crater site, 7: Laptev Sea site. Bathymetry was obtained from IBCAO [33].

We investigated the question of species identity of frenulates at two Arctic seep sites in the Barents Sea, where frenulates have been observed but not identified. These two sites are a cluster of gas hydrate-bearing mounds/pingos in the Storfjordrenna trough (76°6.55’N 16°0.23’E, 380 m water depth) [29–31], and a complex of craters and pingos in the Bjørnøyrenna (Bear Island) trough (74°55.04’N 27°46.18’E, 350 m water depth, Fig 1) [32]. We used light and electron microscopy to undertake a detailed morphological investigation, including comprehensive comparisons with both *O*. *haakonmosbiensis* and *O*. *webbi*. Furthermore, we enhanced our species identification using DNA taxonomic methods and we extended our molecular analysis to samples identified through morphology alone, as being *Oligobrachia haakonmosbiensis* [13] thereby providing the first molecular framework for *Oligobrachia* species identity from high latitude Atlantic and Arctic seeps. In addition to characterizing the frenulate hosts, we also identified their bacterial symbionts using comparative sequence analysis of the 16S rRNA gene, as well as visualizing and localizing them within animal tissue through symbiont-specific fluorescence *in situ* hybridization (FISH) probes and transmission electron microscopy. The presence of bacterial genes involved in thiotrophy and methanotrophy was investigated using dedicated primer sets.

## 2. Methods and materials

### 2.1 Study sites

#### 2.1.1 The Storfjordrenna pingos

**Fig 2:**
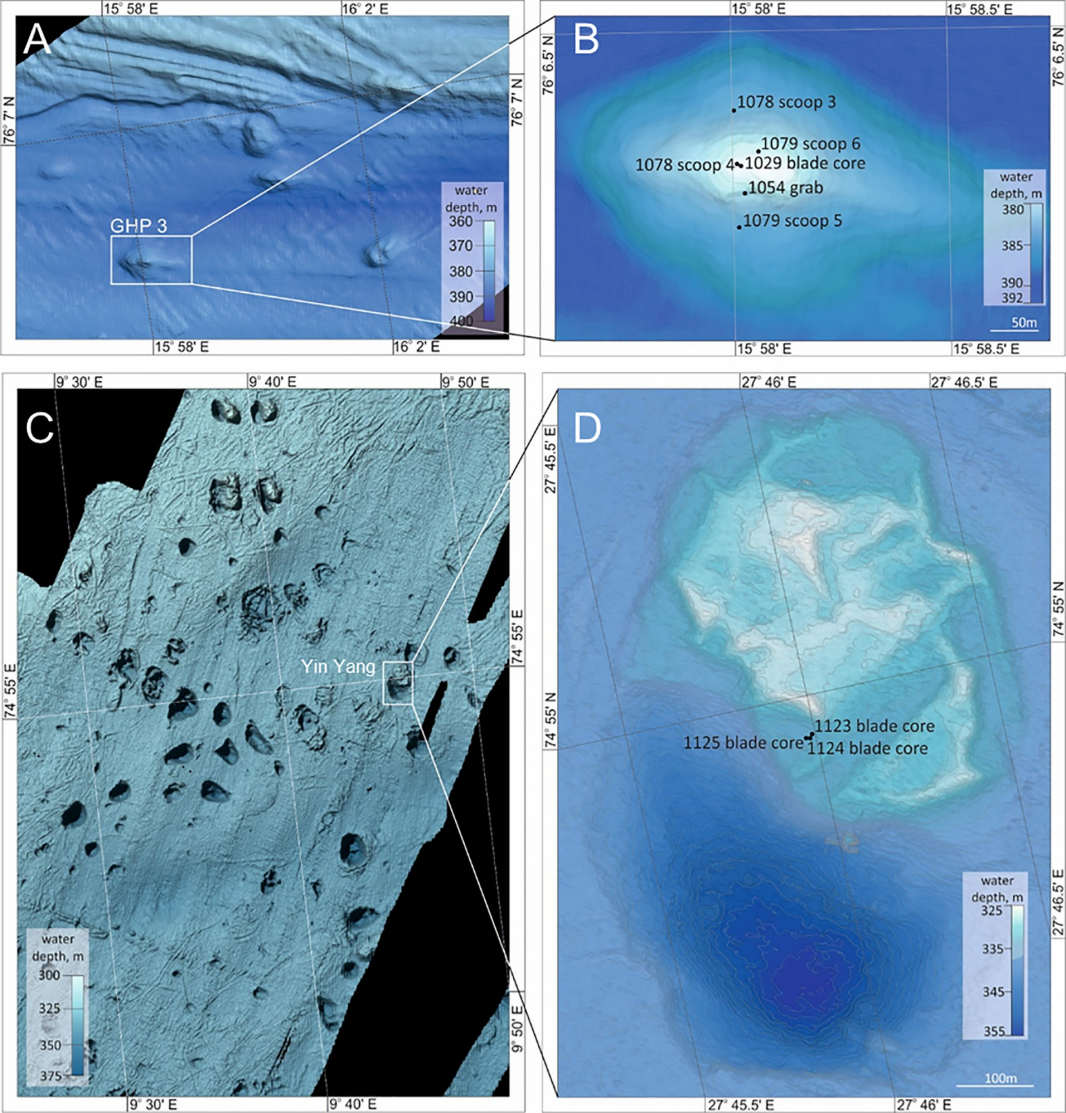
The pingo and crater sites. A: overview of the pingo site (location 5 in Fig 1). Individual pingos are visible in the bathymetry as raised bumps on the seafloor. B: Gas hydrate pingo 3 (GHP3) with sampling locations marked with black dots. C: overview of the crater site (location 6 in Fig 1). D: the Yin Yang pingo-crater complex from the crater site, with sampling locations marked.

South of Spitsbergen, on the Storfjord cross shelf trough, at a depth of about 380 m, an area with numerous domed, sub-circular features from which gas hydrates were recovered in sediment cores, was discovered recently [30,32] (Figs 1 and 2). The seabed mounds, or gas hydrate pingos at this site (referred to here as the ‘pingo site) are about 280–450 m in diameter and rise 8–12 m in height above the seafloor (Fig 2). Seismic surveys revealed gas accumulation in the underlying subsurface and acoustics surveys recorded free gas emissions into the water column from the summits of most of the pingos [29]. Measurements from seafloor sediments indicated gas composition to be primarily methane of thermogenic origin [29]. Worm samples were collected from a single pingo feature at this site (GHP3, Fig 2).

#### 2.1.2 Bjørnøyrenna crater area

The Bjørnøyrenna crater area (referred to in this study as the ‘crater site’) is located about 350 km southeast of the pingo site, in the northern part of Bjørnøyrenna (Bear Island Trough, Fig 1). The site consists of an area of about 440 km^2^ at ~350 m water depth, containing more than a hundred large, 300–1000 m wide, 30 m deep craters, cut into the sedimentary bedrock, with equally wide, 20 m high mounds (pingos) often occurring on the flanks [34,35] (Fig 2). Echosounder profiles revealed hundreds of gas flares rising to about 200 m into the water column throughout the site, originating from the craters or from the mounds [32]. Similar to the pingo site, free gas accumulations were also visible in the sub-surface seabed below the craters. Measurements revealed the gas consists primarily of methane of thermogenic origin [32]. For this study, one crater-pingo complex was sampled (named Yin Yang, Fig 2).

#### 2.1.3 Other sampling locations

In addition to frenulates from the pingo and crater sites, samples from a few other sites in the Arctic and the Norwegian Sea were included in our DNA analysis of host species. Particularly, samples from a number of sites used by Smirnov [13] in his revision of *O*. *haakonmosbiensis* were used in order to add a molecular perspective to the current morphology-based evaluation of *Oligobrachia* frenulates at Arctic and high latitude Atlantic seeps. These sites are: the Nyegga/Storegga slide in the Norwegian Sea, a shallow seep site in the Laptev Sea, HMMV (sites in Smirnov’s revision [13]), plus a seep site within a seafloor canyon off the coast of the Lofoten islands (see overview of all the sampling sites in Fig 1). The sampling of frenulates at Nyegga was conducted on a gas hydrate pingo around a pockmark named G11 [36] (64°40.00’ N, 5°17.35’ E) at about 735 m water depth on the continental shelf above the Storegga slide. Samples from HMMV are a subset of the original samples used to first describe the species [12] and were collected from vast worm fields at a water depth of about 1260 m (72°00.34’ N, 14°42.76’ E). Sampling at Nyegga and HMMV took place in May-June 2006, during the VIKING cruise [37]. The Lofoten canyon samples were collected in August 2017 at a site of active methane seepage in a canyon system off the Lofoten islands of Norway at a depth of about 750 m (68°9.43’ N, 10°27.95’ E). The Laptev Sea samples were collected in 2017 during the 69^th^ expedition of the R/V *Akademik Mstislav Keldysh* at Station 5623 (76°53.68’ N, 127°48.16’ E), at a depth of 63 m. At all sites excluding the Laptev Sea, where a 2 m Sigsby trawl was used, samples were usually collected with remotely operated vehicles.

#### 2.1.4 Sample collection and on board fixation

Samples were collected from the pingo and crater sites in June 2016 (cruise number CAGE 16–5) aboard the R/V *Helmer Hanssen* (UiT, The Arctic University of Norway), from locations where video surveys showed the presence of numerous, large, dense fields of siboglinid worms. At the pingo site, worms were collected with a blade core (sample number 1029) deployed with the ROV 30K (Norwegian Institute of Science and Technology). In addition to the ROV-operated blade core, worms were collected from GHP3 via a Van Veen grab operated from the ship. This grab is operated directly from the ship and an image of the sampling location is not available. The location was decided based on gas flares visible on the echosounder. Most of the grab sample was sieved for quantitative community analysis. This process had the potential to severely damage the delicate siboglinid worms, therefore a miniature push core (5 cm diameter) was carefully inserted into the grab sample to remove the worms without damaging them (sample number 1054). Additionally, a number of sediment scoops were taken at GHP3 in order to collect and quantify epifauna and infauna (samples 1078 and 1079). This resulted in the collection of large numbers of worms from which a number of individuals were removed and processed for this study.

At the crater site, the crater-pingo complex, named ‘Yin Yang’, was sampled (Fig 2). Video surveys of the Yin Yang system revealed large aggregates of siboglinid worms in the pingo part of the complex. One push core and two blade cores were collected from ‘Yin Yang’ (see Fig 2): 1124 (push core), 1123 (blade core) and 1125–3 (blade core). The core locations were chosen based on the visible presence of siboglinid worms. In some cases, such as for cores 1123 and 1125–3, the worms appeared white and ‘fluffy’ at their anterior ends, which was caused by the presence of abundant bacteria attached to the outer surface of the tubes (S1 Fig for visuals on all ROV sampling locations and S2 Fig for view of tube epibacteria). At the crater site, within the 1124 sample collection, one individual of *Spiochaetopterus* sp. (Annelida, Polychaeta, Chaetopteridae) was found. Since this is a tube dwelling worm not known to contain endosymbiotic bacteria, and at the site was exposed to similar conditions as the focus frenulate worms, it serves as a good control, particularly for the visual based FISH analysis. Therefore it was treated and preserved similar to the frenulates in this study.

All core samples were rinsed with cold, filtered seawater. Individual worms were extracted from their tubes by holding down the posterior end of the tubes with a lightweight pair of forceps, and gently squeezing the tube behind the worm with a fine paintbrush. To ensure that host and symbiont based analyses were conducted on the same individuals, a single individual was divided into pieces, each for the different fixations outlined below [38]. The extraction process was difficult, and whole worms or even large pieces were not always obtained. Therefore, all fixations were not carried out on all individuals. Samples were processed within 6–8 hours of recovery. In the meantime, the samples were stored in chilled, filtered sea-water in the dark.

Trophosome (host and symbiont) and frenulum (host) tissue was preserved in 100% ethanol for DNA analyses. Trophosome tissue was additionally fixed for FISH analysis as follows: 4 hours in 2–4% formaldehyde (with filtered seawater) at 4°C, removal of formaldehyde through two rinses in filtered seawater, and a three-step ethanol dehydration series (50%, 70%, 80%, 20 minutes each). Samples were then stored in 80% ethanol.

Other body sections were preserved for electron microscopy. This consisted of an initial fixation, for 4 hours with 4% glutaraldehyde in a phosphate buffered saline (PBS) solution (corrected to 0.1M). Samples were then rinsed and stored in filtered seawater containing sodium azide (0.13 g for 50 ml) until post-fixation.

In addition, a number of individuals were stored in 4% formalin, to serve as reference samples for morphological and anatomical investigations.

### 2.2 DNA analyses

DNA from all samples was isolated using the DNEasy Blood and Tissue kit (Qiagen, Germany) according to the manufacturer’s instructions. DNA quality and quantity were determined by 1% agarose gel electrophoresis and a microspectrophometer (NanoDrop).

DNA from the trophosome tissue of the crater worms was used for sequencing of the 16S rRNA gene of bacterial symbionts and for amplifying fragments of functional genes. Genes encoding adenosine -5’ -phosphosulfate (APS) reductase (*aprA*) and particulate monooxygenase (*pmoA*) were targeted as indicators of sulfur and methane oxidation, respectively. Bacterial 16S rRNA genes of the pingo and crater worms were amplified using 30 PCR cycles (94°C for 45 sec; 48°C for 45 sec and 72°C for 1 min 45) with primers 27F (5’-AGA GTT TGA TCA TGG CTC AG-3’) and 1492R (5’-GTT ACC TTG TTA CGA CTT-3’) [39]. The primers used for *aprA* were APS1-FW (5’-TGG CAG ATC ATG ATY MAY GG-3’) and APS4-RV (5’-GCG CCA ACY GGR CCR TA-3’) and amplification was run for 30 cycles (94°C for 1 min; 58°C for 1 min and 72°C for 1 min) [40]. Primers A189F (5’-GGN GAC TGG GAC TTC TGG-3’) and M661R (5’-CCG GMG CAA CGT CYT TAC C-3’) were used for *pmoA* and amplification was run for 30 cycles (94°C for 1 min; 55°C for 1 min and 72°C for 1 min) [41]. All PCR products were purified via the QiaQuick PCR Purification kit (Qiagen, Germany), ligated with the TOPO TA vector, and transformed to *E*. *coli* TOP10 cells (Invitrogen) as per the manufacturer’s recommendations. Inserts from plasmids were sequenced at the GATC facility (28 clones per investigated specimen, Sanger sequencing). For each gene, the obtained sequences were added to a dataset consisting of best blast hits as well as representative sequences, and aligned using Muscle [42]. Alignments were visually checked.

**Fig 3:**
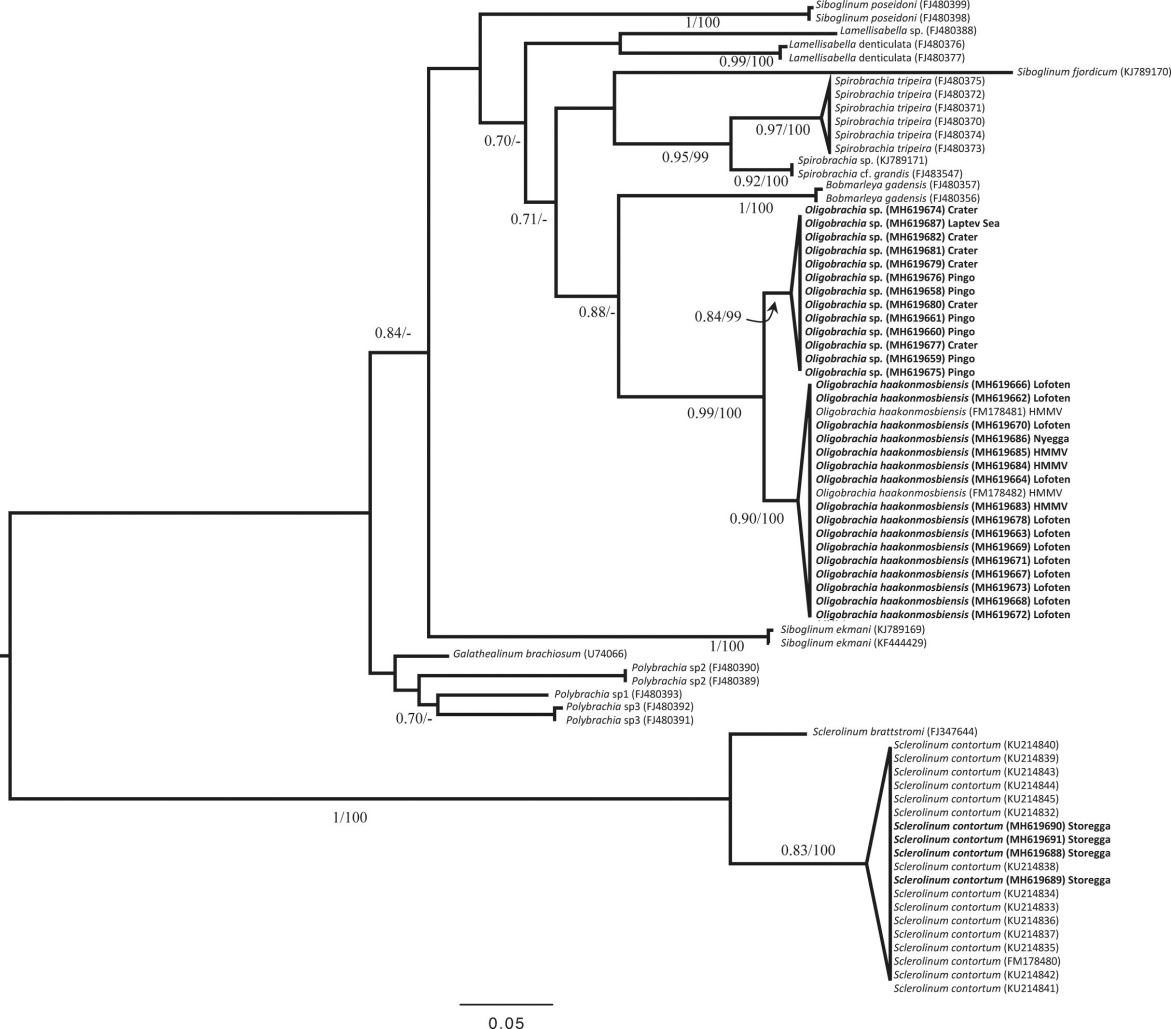
mtCOI tree. Maximum likelihood (ML) tree obtained with PhyML on a 473-bp alignment of a fragment of the mitochondrial COI gene for frenulate Siboglinidae, with a SPR-tree-searching approach. The moniliferan siboglinid *Sclerolinum* spp. was used as the outgroup. For clades with specimens from different geographic origins, the area of capture is indicated. The accession numbers are provided for each sequence in parentheses after the sample number. Branch support: left = approximate likelihood ratio test ALRT; right = bootstrap (%) with the Kimura-2-Parameter distance method. ‘-‘ indicates incongruence of topologies. Only nodes with aLRT values greater than 0.7 are indicated.

A fragment of the mitochondrial COI was used to investigate host genetic relationships of samples from all the sampling sites (pingo site, crater site, Nyegga, Lofoten canyons, HMMV and Laptev Sea). The ‘universal’ primers HCOI2198 and LCOI1490 were used for the barcode approach [43] (35 cycles at 94°C for 1 min; 52°C for 1 min and 72°C for 1 min). Positive amplification was checked on a 1% agarose gel with GelRed to visualize DNA under UV light. Sequencing of the PCR product with the PCR primers was outsourced to Eurofins Scientific. Both strands were sequenced and the assembled resulting sequence was manually curated, if necessary. Sequences from other frenulate Siboglinidae were used to provide a phylogenetic context (see Fig 3 for accession numbers). Moniliferans (*Sclerolinum brattstromi* and *S*. *contortum*) were used as outgroups.

Phylogenetic reconstructions based on nucleotide sequences were performed using a Maximum Likelihood approach under a General Time Reversible model with variable evolutionary rates among sites (gamma distribution) and invariant sites (GTR+G+I, model selected by iModelTest 2.1.10), using the software MEGA 7 [44] and PhyML, with a Subtree-Pruning-Regrafting tree searching approach. Branch support was evaluated with an approximate likelihood ratio test (aLRT). Analysis of the alignment in DAMBE6 did not reveal significant substitution saturation [45]. For the host tree, a distance tree based on Kimura-2-Parameter distance was established with 1000 boostrap replicates and the branch confidence values are also reported on the ML tree.

The haplotype network for the host was obtained and edited with PopART [46] with a TCS approach [47]. The sequences were provided with location as a trait to allow a representation with geography as a possible underlying factor. The Automated Barcode Gap Detection (ABGD) [48] was used to test whether the divergence in specimens of the *Oligobrachia* clade (see Results) was consistent with the existence of several species.

### 2.3 FISH

Samples from the pingo and crater sites were processed for FISH analysis by embedding trophosome fragments into Steedman’s wax, consisting of polyethylene glycol distearate and hexadecanol in a 9:1 ratio by volume. Cross sections of 4–7 **μ**m thickness were cut using a microtome (Thermo). After sectioning, the wax was removed with ethanol and sections were rehydrated through a decreasing ethanol series. Hybridization was performed at 46°C for 3 hours in a buffer containing formamide, then washed with a buffer according to the protocol in Duperron et al. [49]. Tissue sections were covered with DAPI-containing SlowFade anti-bleaching reagent and a coverslip (Thermo Fisher Scientific). Slides were kept in the dark and cold until observation under a microscope. The details of the probes, targets and the associated formamide concentrations are listed in Table 1. The general probes used were EUB338, which targets most Eubacteria [50], GAM42, which targets Gammaproteobacteria [51], and EPSY549, which is a probe targeting Epsilonproteobacteria [51]. The NON338 probe (antisense of EUB338) was used as a negative control [52]. Symbiont-specific hybridizations were carried out using probes LaSp60 and LaSp64, which were designed by Duperron et al. [49], based on probes from Lösekann et al. [11]. The probes used by Lösekann et al. [11] were designed specifically for the symbionts of the frenulate worm *Oligobrachia haakonmosbiensis*. The LaSp probes used in this study are a modification of these probes and less specific, thereby targeting sulfur-oxidizing symbionts from siboglinids including both vestimentiferans and frenulates. However, co-hybridization of both probes is specific for symbionts displaying identical sequence to that of *O*. *haakonmosbiensis* in the 16S rRNA regions targeted by the probes. An Olympus BX61 epifluorescence microscope with DAPI, Cy-3 and Cy-5 filters (Olympus, Japan) was used to visualize hybridized sections, and images were acquired using a SP5 confocal microscope (Leica, Germany). In addition to the standard negative FISH controls with the samples themselves (i.e. NON338 probe, and leaving out probes for at least one section per slide), an additional negative control was added to the study by applying the FISH procedure to a *Spiochaetopterus* sp. worm found at the crater site. This worm is conventionally heterotrophic and is not known to contain internal bacterial symbionts. It therefore served as an additional control in our experimental setup for the specificity and fluorescence of our probes.

**Table 1:** Probe details.

Probe	Sequence (5' - 3')	% formamide	Target	Reference
EUB338	GCTGCCTCCCGTAGGAGT	30–50	Most eubacteria	Amman et al., 1990
LaSp60	CCATCGTTACCGTTCGAC	30	Siboglinid endosymbionts	Duperron et al., 2009
LaSp640	CACACTCTAGTCAGGCA	30	Siboglinid endosymbionts	Duperron et al., 2009
GAM42	GCCTTCCCACATCGTTT	30	*Gammaproteobacteria*	Manz et al., 1992
EPSY549	CAGTGATTCCGAGTAACG	50	*Epsilonproteobacteria*	Manz et al., 1992
NON338	ACTCCTACGGGAGGCAGC	30	Negative control	Wallner et al., 1993

Oligonucleotide probes used for FISH analyses in this study and formamide concentrations.

### 2.4 Microscopy

#### 2.4.1 Optical microscopy

A Leica DFC 320 camera mounted on a Leica MZ6 dissecting microscope was used to take images of live animals from the pingo and crater sites at sea, when conditions permitted. These images however, did not comprehensively cover every body part of every individual, due to difficulties in to taking images aboard a moving ship. The animals preserved for microscopy were photographed before embedding to allow for the examination and measurement of morphological features. An Olympus SZX9 stereozoom dissecting microscope fitted with an Olympus E450 digital camera was used for the optical imaging of the fixed individuals. Specifically, comparisons were made with *Oligobrachia haakonmosbiensis* and *Oligobrachia webbi* as per Table 2 in Smirnov [13], which covers all the different visible body parts and cuticular plaques.

**Table 2:** Morphological features of *O*. *haakonmosbiensis*, *O*. *webbi* and the pingo and crater worms.

Feature	*O*. *haakonmosbiensis*	*O*. *webbi*	crater/pingo worms	confirmed/measured with (n)
**Tube, diameter (mm)**	**0.55–0.9**	**0.5–0.68**	**0.44–0.95**	**live pictures (12)**
Tube, color	brown-black	brown-black	brown-black	live pictures (all)
Tentacles, number	5–12	6–8	5–9	live pictures (15)
Tentacles, pigment	yes	yes	yes	live pictures (all)
Pinnules	no	no	no	live pictures, SEM (all)
**L**_**cl**_**/D**_**f**_ **(cl = cephalic lobe, f = forepart)**	**0.91**	**0.49**	**0.36–0.98 (average 0.63)**	**live pictures (19)**
s^1^ (groove between cephalic lobe and rest of 1st segment)	yes	yes	yes	live pictures, SEM (all)
s^2^ (groove between 1st and 2nd segments)	no	sometimes	yes	live pictures, SEM (all)
**Forepart (diameter)**	**0.45–0.7**	**0.25–0.4**	**0.24–0.77**	**live pictures (19)**
*Forepart*, *glandular patches****+***	*cl spot*, *bands*	*bands*	*bands*	live pictures (all)
*Bridle keels*, *color****+***	*thin*, *brown black*	*thin*, *brownish*	*thin*, *brown black*	*live pictures (all)*
Bridle keels, fused dorsal	sometimes	sometimes	yes	live pictures, SEM (16)
Bridle keels, fused ventral	no	no	no	live pictures, SEM (8)
*Trunk*, *metameric part*, *glandular patches+*	spots, bands	spots, bands	spots, bands	live pictures (all)
Trunk, anteriormost part, rows of pyriform glands	3–4	3–4	4	live pictures, SEM (all)
**Trunk, metameric part, cuticular plaques, diameter (μm)**	**20–24**	**23–30**	**16–30 (average 22)**	**SEM (9)**
**Thickened zone, papillae**	**14–22, 2 rows**	**15–26, 2 rows**	**12–14, 2 rows**	**live pictures, SEM (3)**
**Thickened zone, cuticular plaques, diameter (μm)**	**36–48**	**28–34**	**16–35 (average 27)**	**SEM (21)**
Postannular papillae in each row	3–5	6–7	5–7	TEM fixed samples, SEM (5)
**Postannular cuticular plaques, diameter (μm)**	**~32**	**35–48**	**29–41 (average 33)**	**SEM (8)**
Girdles region, papillae (number)*	**p**^**2**^**(6–8), p**^**3**^**(1)**	**none**	**p**^**1**^(1–2)*, p^**2**^**(8), p**^**3**^**(2)**	**SEM (3)**
Girdles region, cuticular plaques, diameter (**μ**m)	36–48	n/a	14–32, average 20	SEM (10)
Anterior girdle fused dorsally	no	no	no	SEM (2)
Anterior girdle fused ventrally	no	no	no	SEM (2)
Posterior girdle fused dorsally	no	no	no	SEM (2)
Posterior girdle fused ventrally	sometimes	sometimes	sometimes	SEM (2)
Girdles, chaetae, rows	2	2–5	2–3	SEM (5)
Girdles, chaetae, size (**μ**m)	14–15	11–20	10–17 (average 13)	SEM (26)
Girdles, chaetae, anterior teeth	yes	yes	yes	SEM (26)
Girdles, chaetae, untoothed zone	no	no	no	SEM (26)
Spermatophore, length (**μ**m)	~700	unknown	unknown	n/a

#### 2.4.2 Electron microscopy

Samples from the pingo and crater sites were rinsed with 0.2 M sodium cacodylate buffer (pH 7.4) twice and then postfixed in 1% osmium tetroxide in cacodylate buffer for one hour in the dark at 4°C. Following this post fixation, the samples were again rinsed four times, at room temperature, in sodium cacodylate buffer and then dehydrated in an increasing ethanol series (30%, 50%, 70%, 100% ethanol). The most intact fragments were selected for scanning electron microscopy (SEM), ensuring that each body part was represented at least once. Following dehydration, the samples were dehydrated in a critical point dryer (BALTEC CPD030). They were then gold coated and observed under a PHENOM G2 PRO scanning electron microscope.

Postfixed samples were embedded in Spurr’s resin or Epon. Ultra-thin and semi-thin sections were made with a Leica Ultracut UCT microtome and diamond knives (Diatome histo-diamond knife for semi-thin 1**μ**m sections). Semi-thin sections were transferred to glass slides and stained with toluidine and azur blue and observed under a Leitz Laborlux D microscope equipped with an Infinity 3 digital camera. Ultra-thin 60 nm sections were transferred to pioloform- or formvar-coated grids and stained with uranyl acetate and lead citrate. The stained ultra-thin sections were then observed with a JEOL 1400 transmission electron microscope.

## 3. Results

### 3.1 Hosts: Molecular data

Mitochondrial COI (473 bp) was amplified and sequenced successfully from six samples each from both the pingo site and the crater site (S1 Table). Among the other sites, COI was amplified and sequenced from 13 samples from the Lofoten canyons, one sample from Nyegga, three samples from HMMV and one sample from the Laptev Sea site.

Although some of the deep nodes in the phylogenetic tree (Fig 3) offer poor support, some nodes display likelihoods greater than 0.8. All specimens of frenulates sequenced in this study form a clade with 0.99 likelihood and 100% boostrap support. The specimens from the Lofoten canyons, HMMV, and Nyegga form a clade with previously published sequences for *Oligobrachia haakonmosbiensis* (branch support 0.88/100). The specimens from the crater and pingo sites, as well as the specimen from the Laptev Sea, form a distinct clade (branch support 0.87/99) that does not include any other published frenulate species, hereafter referred to as the CPL-clade (Crater-Pingo-Laptev clade).

**Fig 4:**
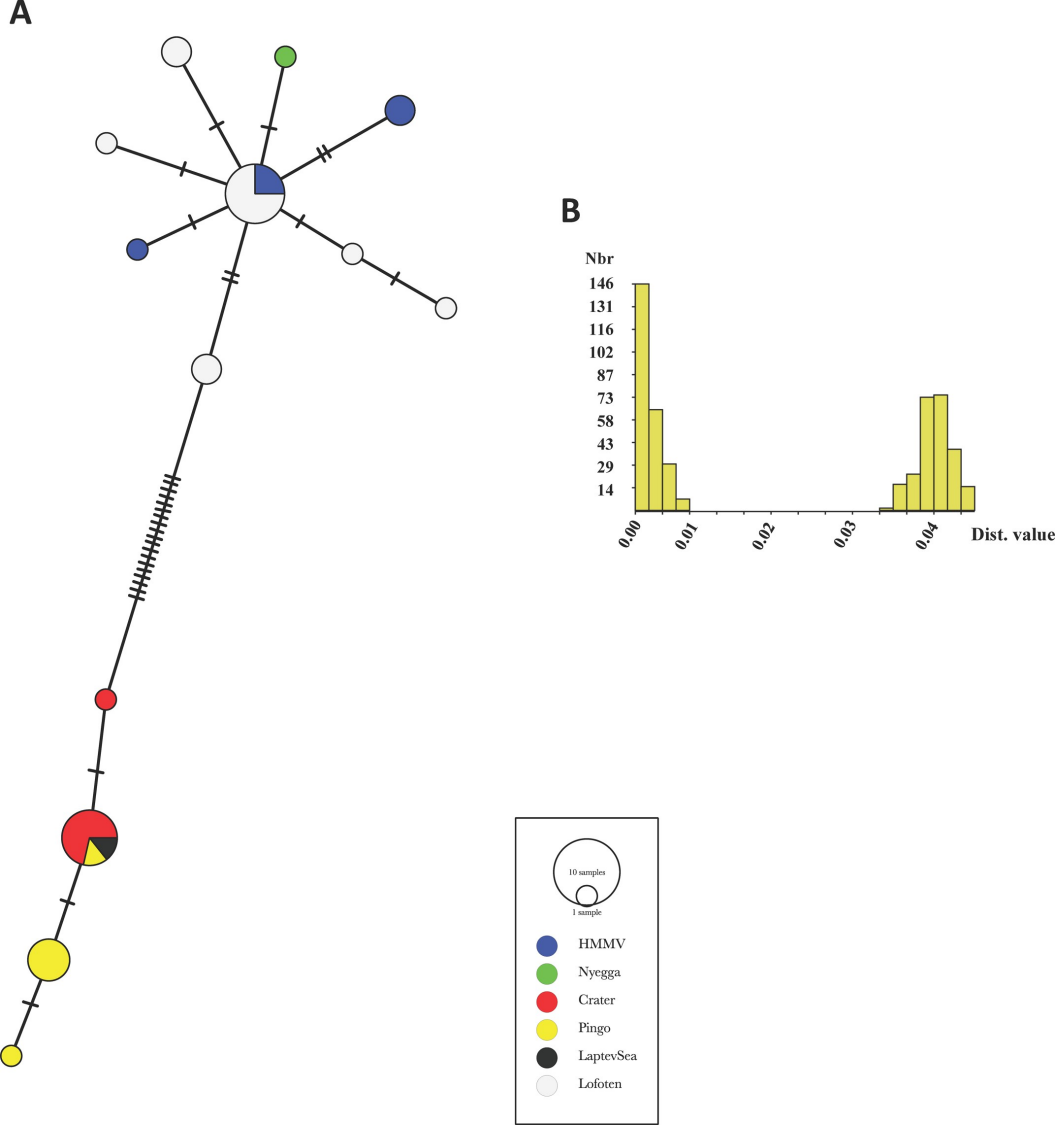
Haplotype network and barcode gap detection. **A:** Haplotype network obtained with a TCS methods in PopART for COI sequences (n = 32) in the *Oligobrachia* spp. clade (see Fig 3). Tick marks indicate mutational steps. B: Automatic Barcode Gap Discovery (ABGD) pairwise distance distribution histogram showing two groups of sequences (prior maximal distance P = 0.00176).

Within each *Oligobrachia* clade, relationships with the geography are not clear and we explored this avenue through a haplotype network and Automatic Barcode Gap Discovery (ABGD) histogram (Fig 4). The sequences segregate into two clusters separated by 19 mutational steps, supporting the dichotomy observed in the phylogenetic tree. This view is supported by the barcode gap observed in the pairwise distances distribution histogram (prior maximal distance P = 0.00176). Intra clade distances varied between 0 and 0.64% (average 0.22 ± 0.17%, n = 246 pairwise distances). In the haplotype network, within each cluster, haplotypes do not segregate according to location, and the most common haplotypes are found in at least two different locations.

### 3.2 Hosts: Morphology

**Fig 5:**
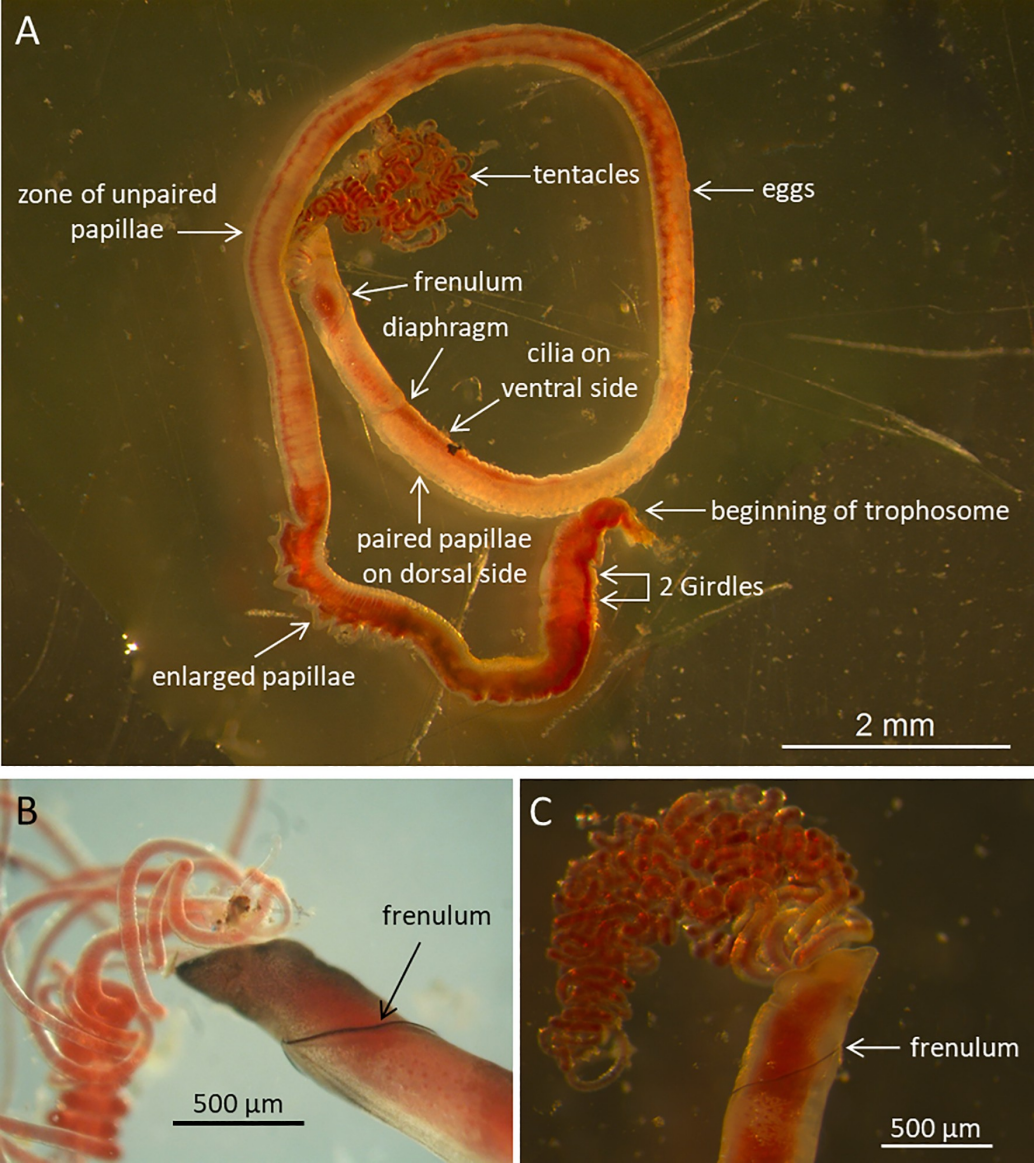
Morphology of the pingo-crater worms and *O*. *haakonmosbiensis*. A: Live profile view of one of the pingo and crater worms (individual #1078–1 from the pingo site). The different body parts are shown (most of the trophosome and the opisthosome are missing). The head is anterior to the frenulum. The forepart is anterior to the diaphragm. The pre-annular trunk is anteriorly limited by the diaphragm, and posteriorly by the two girdles. The post-annular trunk starts with a narrowing behind the girdles and posteriorly contains the black trophosome. B: Close up view of the head and forepart of *O*. *haakonmosbiensis* (Storegga) showing the bridle keels of the frenulum. C: Close up view of the head and forepart of a pingo-crater worm (individual # 1125–7 from the crater site) showing the bridle keels of the frenulum.

**Fig 6:**
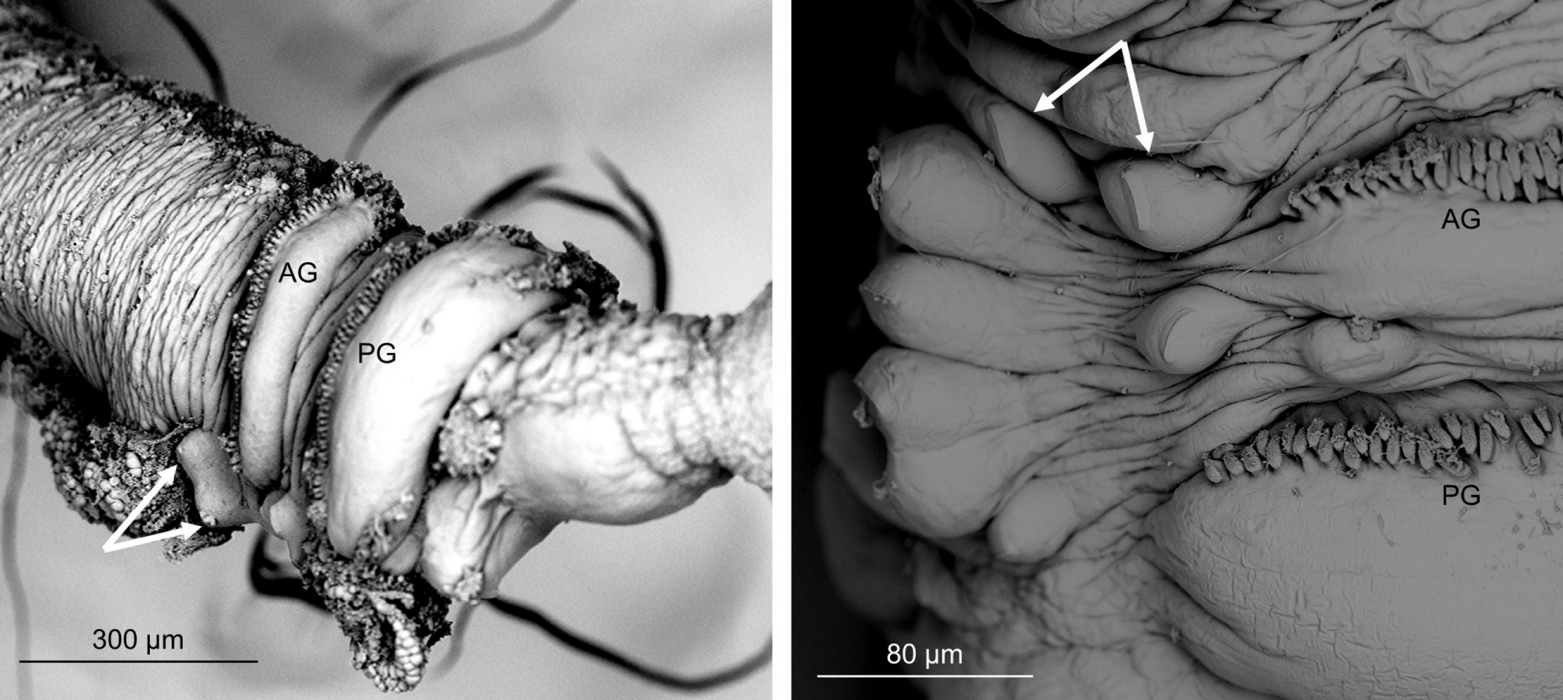
Enlarged papillae of pingo and crater worms. Scanning electron micrographs of the feature that could possibly be unique to the pingo and crater worms: enlarged papillae before the anterior girdle. Arrows indicate the papillae. A line of multi-toothed chaetae is visible just anterior to the each girdle. AG: anterior girdle, PG: posterior girdle.

Morphologically, the pingo and crater worms are very similar to both *O*. *haakonmosbiensis* and *O*. *webbi*. Table 2 lists the features and measurements of the crater and pingo specimens in comparison with *O*. *haakonmosbiensis* and *O*. *webbi*. The features that Smirnov [13] used to distinguish *O*. *haakonmosbiensis* from *O*. *webbi* are highlighted in bold. These are: 1) larger tube diameter in *O*. *haakonmosbiensis*, 2) larger cephalic lobe to forepart diameter ratio in *O*. *haakonmosbiensis*, 3) larger metameric trunk cuticular plaques in *O*. *webbi*, 4) more enlarged papillae in *O*. *webbi*, 5) larger enlarged papillae cuticular plaques in *O*. *haakonmosbiensis*, 6) larger postannular cuticular plaques in *O*. *webbi*, 7) larger chaetae in *O*. *webbi* and 8) the presence of enlarged papillae in the girdle region of *O*. *haakonmosbiensis* [13]. Among the first seven, the pingo and crater worms do not follow the patterns of either *O*. *haakonmosbiensis* or *O*. *webbi*. Instead, they display wider ranges in the sizes of these features than either *O*. *haakonmosbiensis* or *O*. *webbi* alone, so that they mostly encompass the size ranges of both these species. Other features that differ between *O*. *haakonmosbiensis* and *O*. *webbi* are the color of bridle keels (black-brown vs. pale brown respectively), or the pattern of multicellular glands being packed more tightly in the former. The pingo and crater worms have brown bridle keels visible in live pictures (Fig 5) and packed multicellular glands on the trunk, but it is difficult to decide whether they follow the pattern of either *O*. *haakonmosbiensis* or *O*. *webbi*. The most easily differentiable feature between *O*. *haakonmosbiensis* and *O*. *webbi* is the presence of enlarged papillae in the girdle region in the former, which are completely absent in the latter. The pingo and crater worms were also seen to have enlarged papillae in the girdle region. However, they have 1–2 enlarged papillae anterior to the first girdle, which could even be different from *O*. *haakonmosbiensis* and therefore specific to the pingo and crater worms (Fig 6).

Main morphological features of the crater and pingo worms, *O*. *webbi* and *O*. *haakonmosbiensis* (Based on Table II from Smirnov et al. [13]. Measurable features that differ between *O*. *haakonmosbiensis* and *O*. *webbi* are marked in bold. Other differentiating features are marked with + and italics. Features that could be distinctive to the crater and pingo worms (different from both *O*. *haakonmosbiensis* and *O*. *webbi*) are marked with *. The papillae in the girdle region are represented by p, (with the number of papillae indicated in parentheses), where p^1^ refers to the papillae just anterior to the front girdle, p^2^ refers to papillae between the two girdles, and p^3^ refers to papillae just posterior to the hind girdle.

### 3.3 Bacterial symbionts and energy source

**Fig 7:**
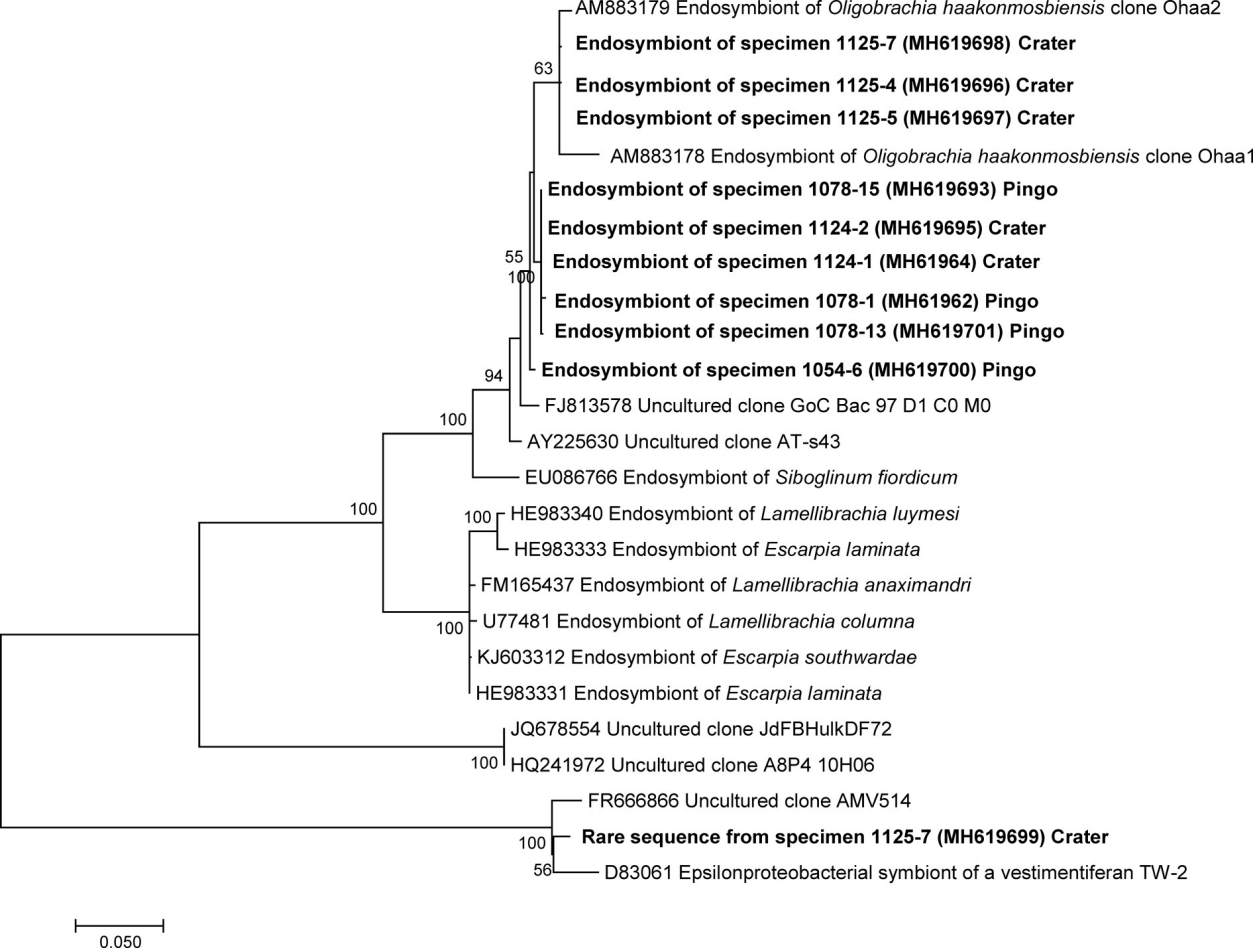
16SrRNA sequence tree. Phylogenetic affiliation of the bacterial symbionts in sampled worms from the pingo and crater sites based on 16SrRNA gene sequences (in bold). A Maximum Likelihood method using the General Time Reversible model with Gamma-distributed rates with invariants was used (1393 positions used). Scale bar represents estimated 5% base substitution. Percentages at nodes correspond to boostrap support values (100 replicates, >50% shown).

**Fig 8:**
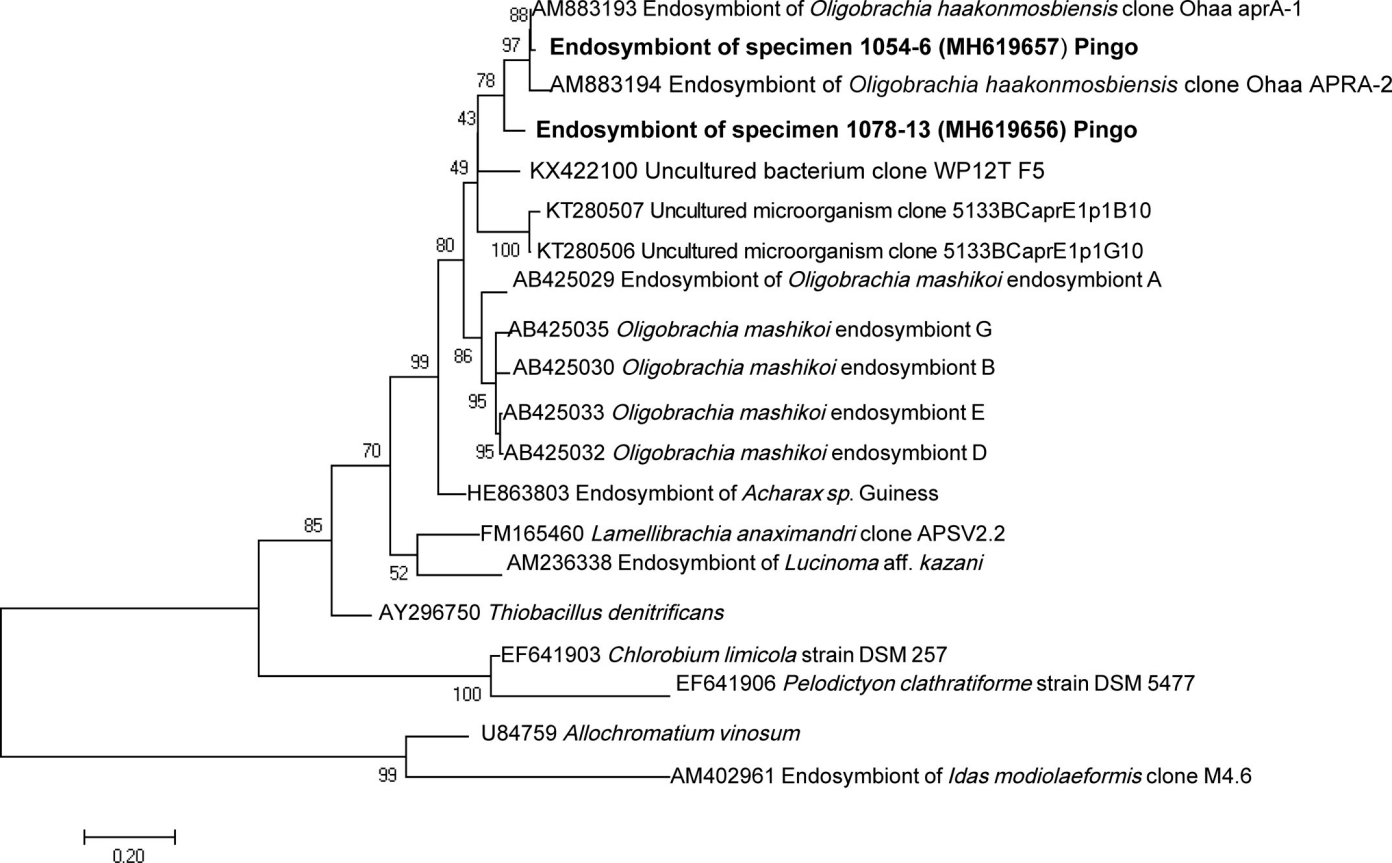
*aprA* sequence tree. Phylogenetic affiliation of APS-encoding genes sequences (*aprA*) from symbionts of *Oligobrachia* specimens 1054–6 and 1078–13 (in bold), inferred using a Maximum Likelihood method and a GTR model (5 categories and invariants, 352 nt positions). Scale bar represents estimated 20% nucleotide substitution. Percentages at nodes correspond to bootstrap support values (100 replicates).

The nine specimens investigated from the crater and pingo sites yielded bacterial 16S rRNA gene sequences, around 1500 bp in length (Maximum Likelihood method using General Time Reversible model with Gamma-distributed rates with invariants was used, 1393 positions, Fig 7). In eight specimens, a single sequence was obtained, likely representing the dominant bacterial phylotype. Specimen 1125–7 displayed both the sequence present in all other specimens, plus a second rare sequence with only 75% similarity. All dominant sequences clustered within a group of Gammaproteobacteria that also included the sulfur oxidizing clones Ohaa1 and Ohaa2 from *Oligobrachia haakonmosbiensis* [11], to which they were highly similar though not strictly identical (Fig 7). The rare sequence from specimens 1125–7 clustered with Epsilonproteobacteria sequences obtained from the tubes of tubeworms and environmental sequences from the Mediterranean Sea [53]. Sequences from specimens 1078–1, 1078–15, 1078–13 (pingos), 1124–1 and 1124–2 (crater site) were almost identical (>99.6% similarity), and those from specimens 1125–4, 1125–5 and 1125–7 (crater site) were also almost identical to each other (>99.8% similarity), though slightly different from the former (<98.4% similarity). These two sequence variants did not correspond with the two different sampling sites (crater vs. pingo). In other words, bacterial symbiont sequences from the pingo frenulates did not cluster separately from the crater samples. The fragment of the gene encoding APS reductase was amplified successfully from the two tested specimens from the pingo site (1054–6 and 1078–13) using a Maximum Likelihood method and a GTR model (5 categories and invariants, 352 nt positions). A single sequence was obtained from each specimen (Fig 8), which exhibited small differences between each other. Both sequences clustered with APS reductase sequences of *O*. *haakonmosbiensis*. The gene encoding particulate methane monooxygenase failed to amplify from all the samples, suggesting it is absent.

**Fig 9:**
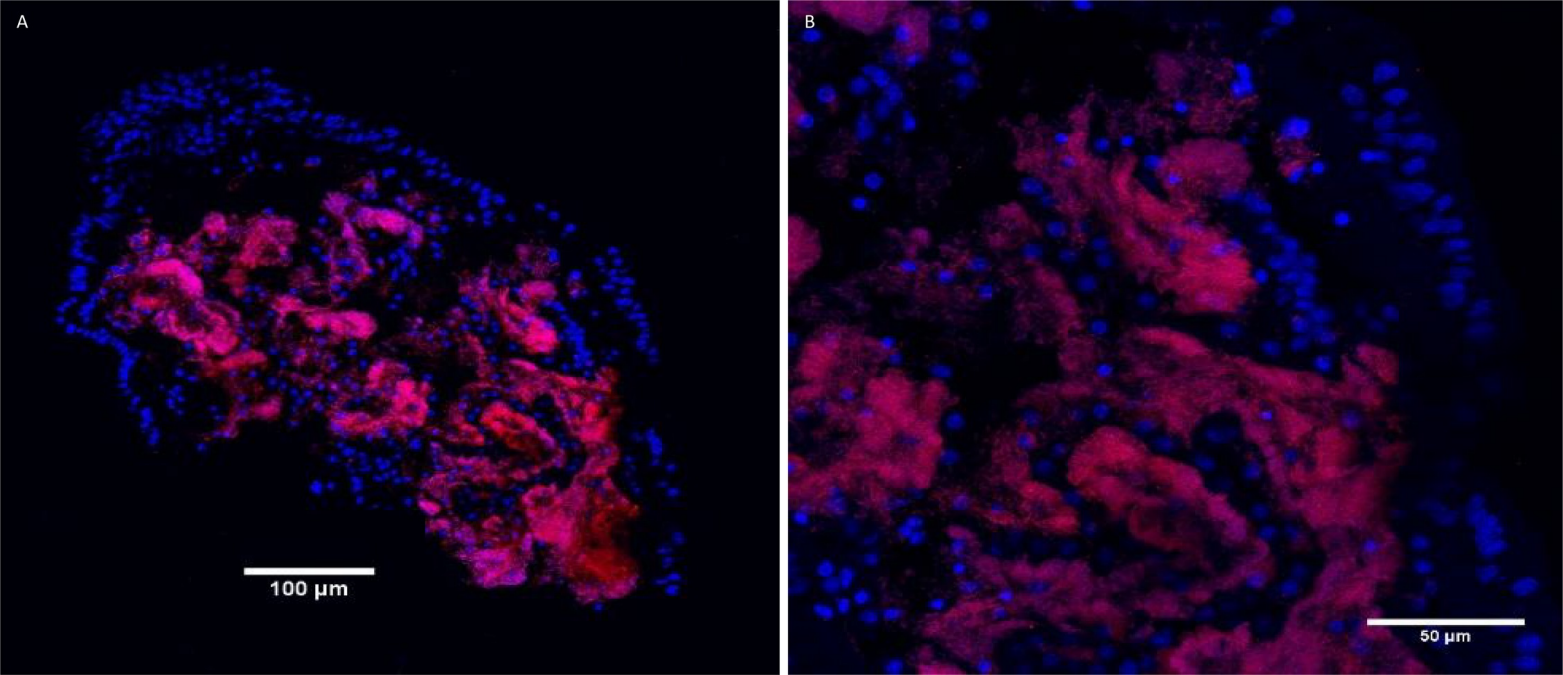
FISH image of pingo worm trophosome cross section. Fluorescence *in situ* hybridization of bacterial symbionts on a cross section of a pingo worm (sample 1078–13): Epifluorescence images showing host nuclei, stained with DAPI (blue), and bacterial symbionts in red, hybridized with LaSp probes from Duperron et al., 2009. A: entire cross section at the level of the trophosome. B: close up of top right part of cross section shown in A. Note the high densities of bacterial symbionts.

**Fig 10:**
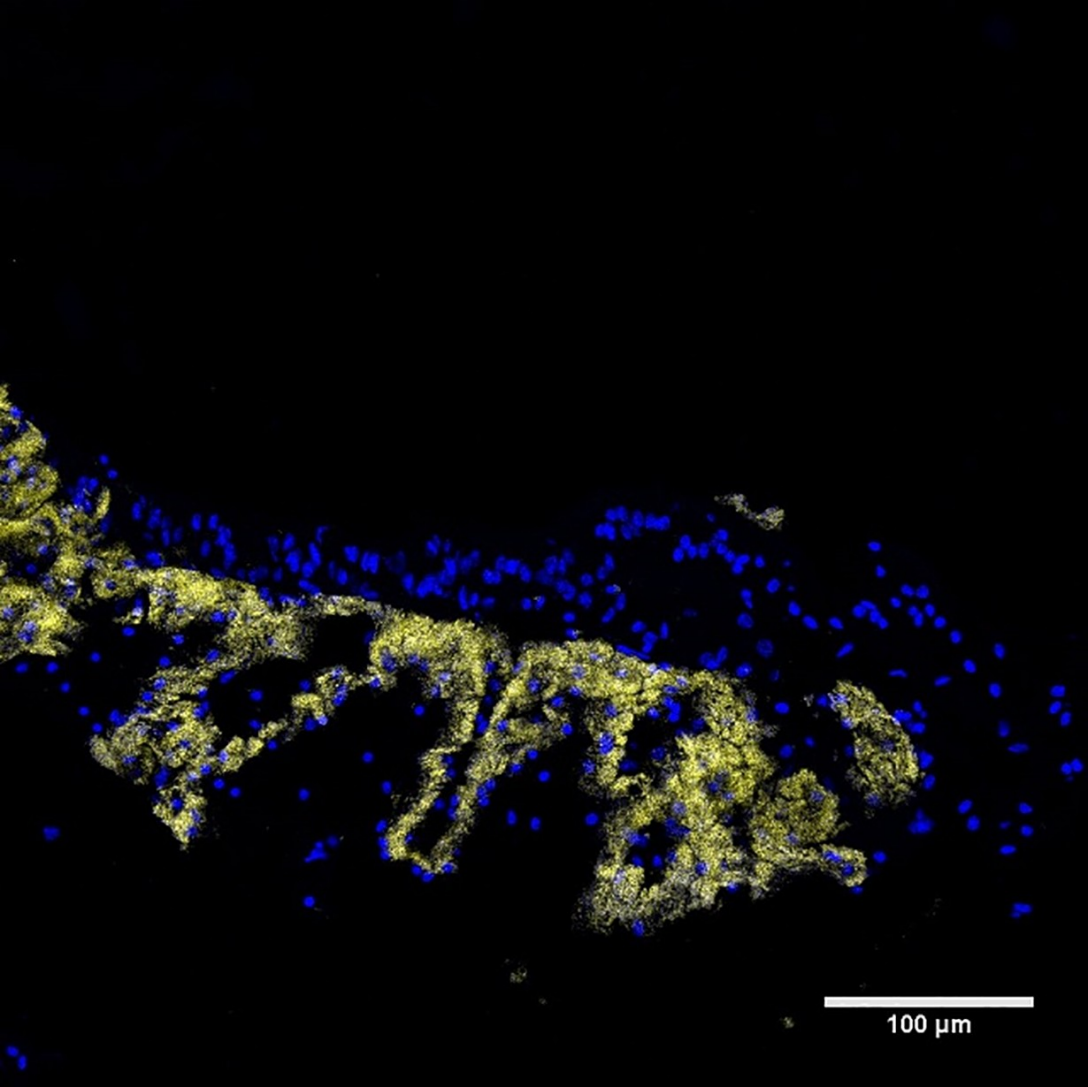
FISH image of crater worm longitudinal section. Epifluorescence image of a longitudinal section of the trophosome of a crater worm (sample 1125–6). Host nuclei appear blue due to DAPI staining. Bacterial cells (yellow) were hybridized with the GAM42 probe. From this point of view, the trophosome appears to contain multiple folds and the bacteria appear to follow these folds.

In correspondence with the 16S rRNA gene sequence results, FISH probes for Eubacteria and Gammaproteobacteria hybridized successfully on all examined sections. Subsequently, probes LaSp60 and LaSp640 (which target all gammaproteobacterial variants from this study) yielded positive co-hybridization results, fully overlapping with the eubacterial signals and thus confirming that most bacteria actually correspond to the dominant recovered 16S rRNA gene sequence. These probes revealed dense and abundant populations of bacteria within the trophosomes of all 4 crater and 3 pingo investigated specimens (S1 Table) (Fig 9). Bacteria tended to be present in folds of the trophosome tissue, and were observed inside the organ (Fig 10). On the other hand, the epidermis and the outer parts of the trophosome only fluoresced with respect to DAPI and worm DNA. The EPSY-549 probe did not hybridize with the bacteria in any of the samples tested, suggesting the absence or rarity of Epsilonproteobacteria. No hybridization, with any of the probes was seen in the non-symbiont containing worm *Spiochaetopterus* sp. (S3 Fig).

**Fig 11:**
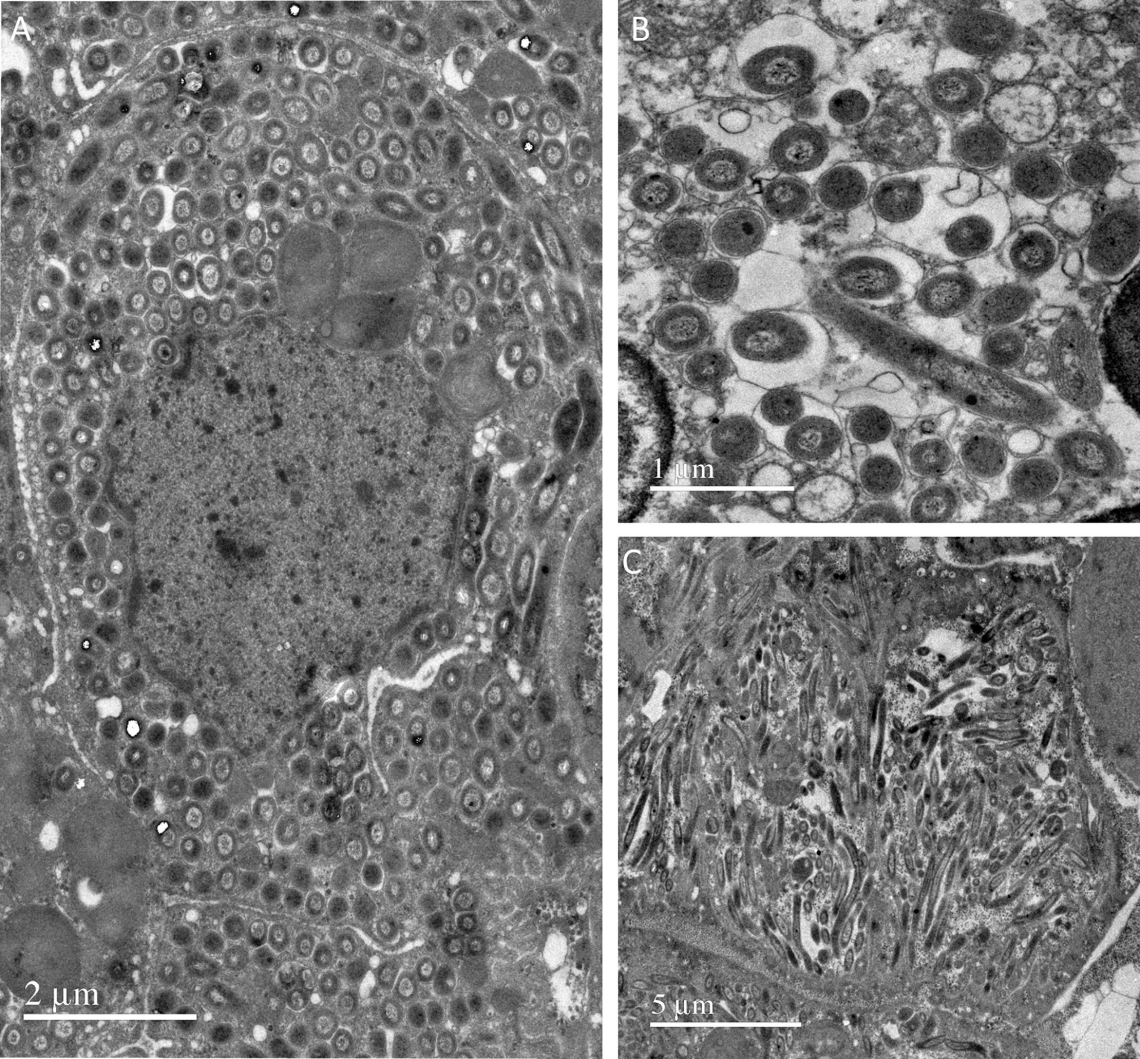
TEM images of pingo and crater worms. Electron micrographs of cross sections of the symbiont bearing trophosome tissue of the pingo and crater worms. Round or rod shaped bacteria were seen, depending on the plane across which the cuts were made, typical morphology of sulfur oxidizing symbionts. A: Entire bacteriocyte densely packed with bacteria. Note the alignment all along a single plane such that they appear circular (cut through cross sections). B: Close up view of the symbionts in cross section. Bacterial cells occur singly, or in pairs or groups of three within individual vacuoles. C. Bacteriocytes where many of the symbionts have been cut longitudinally, showing the rod shaped morphology.

The host cells in the TEM cross sections appeared packed with bacterial cells. In both the crater and pingo worms, the bacterial cells were small, and either round or rod-shaped based on the angle of cut, a morphology that is consistent with sulfur oxidizing symbionts (Fig 11). The diameter of the bacteria ranged between 0.3 and 0.5 **μ**m in cross section, and their length ranged from 2 to 4 **μ**m. The bacteria were located within vacuoles, sometimes singly, but often in pairs or even groups of three or more cells (Fig 11A and 11B), and each bacteriocyte was filled with these vacuoles. The bacteria were all arranged in the same orientation, and either appeared all transversally (Fig 11A) or all longitudinally sectioned (Fig 11C). No bacteria with internal stacked membranes, the usual morphology of methane oxidizing symbionts, were seen in any of the electron micrographs.

## 4. Discussion

### 4.1 Pingo and crater worms are genetically distinct from *Oligobrachia haakonmosbiensis*

There are currently 11 species of *Oligobrachia* described, and among them, *O*. *haakonmosbiensis* and *O*. *webbi* share the unusual characteristic of lacking pinnules on their tentacles. COI data in public databases exists only for *O*. *haakonmosbiensis*, limiting the interpretation of our molecular work. However, data from other genera are available, which allowed us to provide a phylogenetic context for the pingo and crater worms. All the sequences acquired for specimens from the pingo and crater sites, as well as those from the other four sites in this study form a distinct clade with very high support (Fig 3). We will hereafter refer to them as *Oligobrachia*. The sister clade to *Oligobrachia* comprises of the two sequences available for *Bobmarleya gadensis*, a species from mud volcanoes in the Gulf of Cadiz [9]. *Oligobrachia* itself splits into two distinct clades, also with high support. One includes two previously published sequences from *O*. *haakonmosbiensis* (from individuals from the type locality) and our sequences from Nyegga and HMMV, indicating that these sites are indeed inhabited by *O*. *haakonmosbiensis*, as described by Smirnov [13]. Specimens from the Lofoten canyons also fall within this clade; this site therefore also appears to be inhabited by *O*. *haakonmosbiensis*. The three other sites included in this study (pingo, crater, and Laptev Sea site), are inhabited by a single genetically distinct species. This interpretation is supported by the haplotype network and barcode gap, and there is no regionalization of the haplotypes (Fig 4). As no sequence is available for *O*. *webbi* (nor any other species of *Oligobrachia*), we cannot assign the pingo, crater and Laptev Sea worms to an existing species, nor to a new species entirely. Instead, we suggest referring these worms as the *Oligrobrachia* sp. CPL-clade.

The two species of *Oligobrachia* do not overlap in geographic distribution, with *O*. *haakonmosbiensis* in the more southern part of the sampled area (Norwegian Sea), while *Oligrobrachia* sp. CPL-clade is more polar. This situation, of a polar *Oligobrachia* species and a separate species in the Norwegian Sea, contrasts sharply with the other siboglinid found at Arctic vents and Norwegian Sea seeps, *Sclerolinum contortum*. Based on COI data, *S*. *contortum* has a very wide geographic range spanning both polar oceans, the Gulf of Mexico [54,55], and with additional sequences in our study, the Storegga Slide (Fig 3). The distribution of the two *Oligobrachia* clades could be driven by currents since Nyegga, the Lofoten canyons and HMMV, i.e., the sites where *O*. *haakonmosbiensis* is present, all lie along the path of the North Atlantic current, while the *Oligobrachia* sp. CPL-clade sites are only minimally exposed to this current, if at all. Depth could represent another major contributing factor. For instance, *Sclerolinum brattstromi* has been described from Hardangerfjorden, a relatively shallow fjord in Norway, well within the reported distribution range of *S*. *contortum* [56,57], and species boundaries of *Lamellibrachia* siboglinids have been shown to be driven by depth in the Gulf of Mexico and the Pacific [4,58–61]. All *O*. *haakonmosbiensis* sequences come from specimens collected at deeper sites on the continental slope (735 m– 1260 m water depth), while all *Oligobrachia* sp. CPL-clade specimens were collected on the shallow continental shelf (63 m– 380 m water depth).

Samples from a shallow site exposed to the North Atlantic current, such as the type locality of *O*. *webbi*, could improve our understanding of the separation of *Oligobrachia* clades. In fact, our CPL-clade samples could very well correspond to *O*. *webbi* (270 m water depth in the Norwegian Sea, Fig 1). Unfortunately, original *O*. *webbi* samples were preserved in formalin in 1966 which does not permit DNA extraction, and two attempts to collect fresh samples from the type locality were unsuccessful in recovering the species [62,63]. It is therefore not currently possible to constrain how water depth and circulation or geography affect speciation within *Oligobrachia*. Interestingly, a similar pattern of confounding variables with respect to frenulate distributions has been observed in the Gulf of Cadiz [10]. Therefore, constraining distributional patterns of frenulates and differentiating between different oceanographic patterns is an open question in frenulate biology, not only in high latitude regions. A growing interest in Arctic ecosystems could lead to new sites being discovered and sampled in various parts of the Arctic, likely allowing this question to be addressed in the future.

In this study, sequences from a single gene, mitochondrial cytochrome c oxidase subunit I (mtCOI), were used for conducting phylogenetic analyses. The mtCOI marker is usually sufficient for detecting different species in polychaetes and frenulates [10,64,65]. Despite these results, one must bear in mind that for closely related siboglinids, mtCOI can be problematic for discriminating between closely related species. For example, morphologically distinct Gulf of Mexico seep vestimentiferans cannot be discriminated by mtCOI alone [58,66]. Therefore, when mtCOI sequence data point towards different species, as is the case in this study, it is likely that it is indicative of actual species differentiation. For a polychaete family (Siboglinidae) in which species such as *S*. *contortum* or *Escarpia* spp. exhibit no differentiation over very wide geographic ranges [54,55,66], the close geographic distribution of animals from the two *Oligobrachia* clades in spite of a 3–4% divergence further supports the existence of two distinct species. The intra-clade divergence for our *Oligobrachia* sequences (0.22%) is similar to that of *Sclerolinum contortum* (average 0.78 ± 0.35%, n = 135 pairwise distances). It is still possible that more species are present, that are not visible through our mtCOI based methodology, and our results could simply be the tip of the iceberg, but the evidence does not support the idea of a single species across the multiple sites included in this study. Our results indicate that Arctic and high latitude north Atlantic seeps are occupied by at least two species of *Oligobrachia* as opposed to one species with a large distribution covering both Arctic and north Atlantic regions, like the widespread distribution of *Sclerolinum contortum* siboglinids [54].

### 4.2 Absence of consistent morphological differences between animals of the two clades

We carried out detailed morphological examinations of the pingo and crater worms to determine whether CPL-clade members can be differentiated from *O*. *haakonmosbiensis* in the absence of molecular analyses. As mentioned above, the distribution of the CPL-clade on the shallow shelf corresponds with the location of *O*. *webbi*. Therefore, another objective was to determine whether the pingo and crater worms could be considered *O*. *webbi*.

In order to do this, identifying features that differ between *O*. *haakonmosbiensis* and *O*. *webbi* is necessary. Some of these features are difficult to examine objectively. For example, in *O*. *haakonmosbiensis*, multicellular glands are ‘packed more tightly’ than in *O*. *webbi* [13], and without clear enumeration, it is not possible to determine if the pingo and crater worms more closely follow the pattern seen in one or the other of these two species. Similarly, bridle keels in *O*. *haakonmosbiensis* are dark brown, as opposed to light brown in *O*. *webbi*. Though the bridle keels of the pingo and crater worms appeared to be brown in color, such appearances can be affected by fixation or lighting and therefore they should be used extremely cautiously as a means of distinguishing between species.

Other differences between *O*. *haakonmosbiensis* and *O*. *webbi* were identified, which can be more objectively examined. These features are mainly variations in sizes or proportions, such as tube or cuticular plaque diameters. These specific characteristics among the pingo and crater worms did not differ from each other, neither did they correspond to either *O*. *haakonmosbiensis* or *O*. *webbi*; instead, they displayed ranges of numbers and sizes that included the ranges of both *O*. *haakonmosbiensis* and *O*. *webbi* (Table 2). On the other hand, the pingo and crater worms do contain enlarged papillae in the girdle region, as does *O*. *haakonmosbiensis*. However, it is possible that the arrangement and numbers of these papillae could be specific. In the pingo and crater worms, 1–2 enlarged papillae are present just anterior to the girdles on the dorsal side and according to Smirnov’s revision of the species, *O*. *haakonmosbiensis* only has papillae between the girdles and posterior to the hind girdle [13]. However, two papillae are shown anterior to the front girdle in Fig 2D of Smirnov’s revision of *O*. *haakonmosbiensis*. Therefore, it is debatable, whether this feature is truly unique to the pingo and crater worms.

This means that the pingo and crater worms cannot be differentiated from *O*. *haakonmosbiensis* based simply on morphology. In fact, samples from the Laptev Sea site, which belong to the same clade as the pingo and crater worms, were used in Smirnov’s taxonomic revision of *O*. *haakonmosbiensis*, which means that members of the *Oligrobrachia* sp. CPL-clade are literally identical to *O*. *haakonmosbiensis*. The other question, of whether the CPL-clade is the same as *O*. *webbi* can be ruled out if the lack of papillae in the girdle region truly is a specific characteristic of *O*. *webbi*. However, the original description of *O*. *webbi* was made literally by piecing together parts of different individuals, which were removed from their tubes post-fixation, a process that is extremely difficult and highly destructive. Due to this, and the lack of DNA sequences on *O*. *webbi*, we prefer to maintain a cautious approach such that the pingo, crater and Laptev Sea worms are referred to as the CPL-clade as opposed to a new species altogether.

Nonetheless, our results indicate that morphology alone cannot be used to identify and differentiate between *Oligobrachia* worms for whom sequences are available. The inadequacy of external morphology for species discrimination, and the presence of two species based on molecular data suggest that a number of cryptic species of *Oligobrachia* inhabit Arctic and high latitude north Atlantic seeps. Cryptic species complexes are in fact, not uncommon among polychaetes [64,67], or among animals from chemosynthetic environments [68–79]. Multiple cryptic species, however, as opposed to a wide-ranging, cosmopolitan species, is a viewpoint that has only gained momentum due to recent approaches that combine both morphological and molecular methodologies [64,79]. Though our results indicate the presence of cryptic *Oligobrachia* species when one considers Arctic and sub-Arctic locations combined, it is still nonetheless possible that one *Oligobrachia* species (the CPL-clade) has a pan-Arctic distribution. Indeed, *Oligobrachia* frenulates have been found on the non-European side of the Arctic, such as in the Beaufort Sea, and mitochondrial COI sequences from these individuals were about 97% identical to those of *O*. *haakonmosbiensis* [18], close to the divergence observed between the two *Oligobrachia* clades in this study (3.1–4.1%). The absence of published sequences from these samples precludes direct comparisons with the members of the CPL clade, and further research is required in order to determine the geographic extent of the CPL clade’s distribution and whether it is present throughout the Arctic. Given that the pingo and crater sites are distant and quite separated from the Laptev Sea, and that other seep polychaetes are known to have large geographic distributions [54,55], it remains a possibility that the CPL clade is widely distributed across northern latitudes, although more comprehensive sampling and research is required to determine whether this is truly the case.

Among both *O*. *haakonmosbiensis* from the Lofoten canyon site and the *Oligobrachia* sp. CPL clade, a bifurcated ‘arm’ bearing oocytes lined on each side by two blood vessels was seen to emerge ventrally from just below the frenulum (S4 Fig). Due to the lack of any visible ruptures in the blood vessels and body of the individuals in which this feature was observed, we initially believed that this structure might constitute a new, undescribed trait characteristic to females. The fact that the oocytes remained firmly attached in place, as opposed to spilling out further suggested that this ‘egg string’ might constitute an actual feature, since in frenulates, large oocytes supposedly lie more or less free in the hind parts of ovaries [80]. It showed a close anatomical link between blood vessels and oocytes. However, semi-thin sections revealed unlined epidermis and an interrupted cuticle at the ‘attachment point’ of the egg string, which indicates that it is an internal organ (the paired ovaries) that was pushed out of the body, probably as the worm was extracted from its tube (S4 Fig). This artifact occurred quite frequently among samples, and should be kept in mind when working with members of *Oligobrachia*.

### 4.3 Symbiont populations

All investigated pingo and crater frenulate specimens were found to host symbionts that are phylogenetically related to sulfur-oxidizing Gammaproteobacteria previously reported in *Oligobrachia haakonmosbiensis* [11]. Interestingly, two slightly different symbiont 16S rRNA phylotypes were found whose distribution among specimens does not match the distinct sampling sites. Despite them being very closely related, physiological differences could exist between the two phylotypes, but it is highly likely that all are able to perform autotrophy based on the oxidation of sulfide. Sulfur-oxidizing symbionts are reported in most documented frenulates, as well as in other siboglinid annelids regardless of whether they are seep (or vent) endemic, and seems to be a common feature of the clade as a whole [1,3,80–83]. There are reports of methane oxidizing symbionts in certain species [84,85], and even in *Oligobrachia* from the Laptev Sea [20], which based on our results, are probably members of the CPL clade. However, in this study, we were only able to find evidence for sulfur oxidizing symbionts in specimens from the pingo and crater sites. It is possible that genes for methane oxidation and visualization of methanotrophic symbionts were simply missed or hidden across our analyses, however, Lösekann et al. [11] obtained similar results, whereby they could find no evidence for methanotrophic symbionts in *O*. *haakonmosbiensis* despite a wide variety of and rather comprehensive array of analyses.

A striking feature of the symbionts of the pingo and crater worms is their density. Actual quantification is admittedly unavailable, but nonetheless, our FISH images reveal much denser bacterial populations in the trophosomes of the pingo and crater worms in comparison to similar images of the trophosome of *O*. *haakonmosbiensis* (Figs 8 and 9) [11]. Therefore, it is possible that despite overall similar morphology, Norwegian Sea and Arctic seep frenulates differ in terms of the size or abundance of internal bacterial populations. This difference could be driven by geochemical conditions, which, in addition to the above discussed factors of depth and ocean currents, could be an additional factor that contributes towards the separation of the two *Oligobrachia* clades. Specifically, higher sediment sulfide concentrations and subsequently higher symbiont dependence could select for denser symbiont populations in the CPL-clade. Indeed, higher sulfide concentrations were measured in sediment porewater samples from the pingo and crater sites (maximum concentrations of 5 and 8 mmol L^-1^ respectively) [30,86] in comparison to HMMV and the Lofoten canyons (maximum concentration of 1.5 and 3 mmol L^-1^ respectively) [87,88].

Dense symbiont populations is not unusual only in comparison with *O*. *haakonmosbiensis*, but within the context of frenulates overall. Despite being conspicuous organs, frenulate trophosomes are nonetheless considered to be rather small, even relative to the overall size of individual frenulates [81]. Bacteriocytes are estimated to occupy less than 10% of the trophosome volume and tend to be limited to peripheral regions [81,89,90]. This is because despite anoxic, reduced conditions, dissolved sulfide concentrations tend to be low in frenulate habitats and it has been suggested that frenulates supplement their chemosynthesis based diet with the uptake of dissolved organic compounds (DOC) from the sediment [91–94] (though frenulates have also been hypothesized to make use of insoluble sulfides in addition to dissolved sulfide) [95,96]. As a result, the common paradigm is that frenulates have sparse internal symbiont populations, particularly in comparison with vestimentiferans.

However, frenulates have not been studied to the same extent as vestimentiferans and detailed examinations of frenulate trophosomes are limited to a few studies [11,81,83,97,98]. Bacterial abundances in frenulates have been seen to vary based on the cells or regions of the trophosome being observed [81,98], or on sex based differences [81]. Our results, of highly dense symbiotic populations might not necessarily represent a major departure from established notions about frenulate biology, but instead, could highlight a line of research that has not been pursued particularly thoroughly. In fact, studies on frenulate symbioses already demonstrate departures from established generalizations. For example, Kim and Ohta [98] dispute the trend of uniform symbionts in frenulates since they observed zonation of frenulate symbionts of various sizes resembling the radial arrangement of different-sized symbionts within vestimentiferan trophosomes [99]. Deguchi et al. [97] expanded upon the schematic of frenulate trophosome structures: they observed 3D ‘leaves’ of bacteriocytes, which might even resemble what was seen in the pingo and crater worms (Fig 10). Furthermore, the endosymbiont population of frenulates might be more diverse than currently estimated. Indeed, Rodrigues et al. [7] found evidence for heterotrophic bacteria in addition to thiotrophic ones within the trophosomes of frenulates from the Gulf of Cadiz. Clearly, frenulate-bacteria symbioses require more attention, and contexts such as sex or location along the length of the trophosome need to be systematically considered. Additionally, our results suggest that the nature of holobiont composition could be mediated by local geochemical conditions, but whether the size of symbiont populations is determined by host species, or whether a single host species can flexibly adjust their endosymbiont populations in response to changing conditions, or alternatively, whether the symbiont-density is strictly dependent of the influx of sulfide to sustain them, remains an open question.

## 5. Conclusion

Overall, our results provide the first molecular insight into the debate of species identity among non-pinnule bearing *Oligobrachia* frenulates. Even though we were unable to determine whether *O*. *haakonmosbiensis* and *O*. *webbi* ought to still be considered separate species or not, we clearly demonstrate that morphology alone is an inadequate means for determining species identity. At least two cryptic species appear to be found at Arctic and high latitude north Atlantic seeps. Despite similar morphologies, the two species appear to differ substantially with respect to sizes of symbiont populations. The presence or absence of specific species could be related to sediment porewater sulfide concentration, and sulfide availability could also be the factor that determines the density of symbiont populations within the trophosome. Further studies, with samples from multiple Arctic seep sites are required to gain a more comprehensive overview of the numbers of species present, and how their distribution relates with environmental factors such as depth, fluid composition or currents. Given the departures displayed by *Oligobrachia* members from other frenulate species in very fundamental aspects of their biology, including symbiosis, and their significance at seep locations, frenulates represent a good target study group for future studies of Arctic cold seeps and reduced habitats.

## Supporting information

S1 Fig Sampling locations. Visual overviews of the sampling locations at the pingo sites (A-E) and crater sites (F-H). Images are video stills from the ROV 30K’s high definition video system. A: sample 1029 (blade core), B: sample 1078 (first scoop), C: sample 1078 (second scoop), D: 1078 (third scoop), E: 1078 (fourth scoop), F: 1123 (blade core), G: 1124 (push core) and H: 1125 (blade core). Arrows are shown to highlight the presence of frenulates on the seafloor. No visuals for sample 1054 are available since this sample was taken with a Van Veen grab operated directly from the ship.
(TIF)

S2 Fig Tube bacteria. Close-up view of dense epibacterial colonies, seen on the anterior ends of the tubes of many individuals of the pingo and crater worms, giving a white, fuzzy appearance in videos and images.
(TIF)

S3 Fig FISH image of non-symbiont hosting worm. Epifluorescence micrograph of cross section of the trunk of a *Spiochaetopterus* worm from the crater site (sample 1124–6, negative control). Host/animal nuclei are stained with DAPI (blue). Hybridization did not occur and no fluorescence for bacterial symbionts were detected.
(TIF)

S4 Fig Bifurcated ‘egg string’ seen among the *Oligobrachia* clade. *Oligobrachia* clade individual with the ‘egg string’ feature. A: Stereozoom view of the two arms of the feature, beginning immediately posterior of the diaphragm on the ventral side of the animal. The string is lined by a continuous blood vessel on either side of which lie the eggs. B: Semithin cross section at the level of the diaphragm, clearly showing the outpocking of this egg string from the inner body with the broken epidermis. C- Close-up view of the egg string showing longitudinal sections of the blood vessels and their close vicinity to the oocytes.
(TIF)

S1 Table Sample list. List of all the pingo and crater samples used in this study. Single individuals were often used for multiple analyses (different segments for different analyses), particularly if large or whole segments could be retrieved from the tubes. In addition to the pingo and crater samples, 13 worms from the Lofoten canyons, 1 from Nyegga, 1 from the Laptev Sea and 4 *Sclerolinum* samples from Storegga were additionally used for DNA analysis of the worms (mtCOI sequencing).
(PDF)
