# Supplementary figures and images for: Cryptic frenulates are the dominant chemosymbiotrophic fauna at Arctic and high latitude Atlantic cold seeps
Source: PLoS One. 2018 Dec 28;13(12):e0209273. doi: 10.1371/journal.pone.0209273 (PMC6310283; doi:10.1371/journal.pone.0209273)

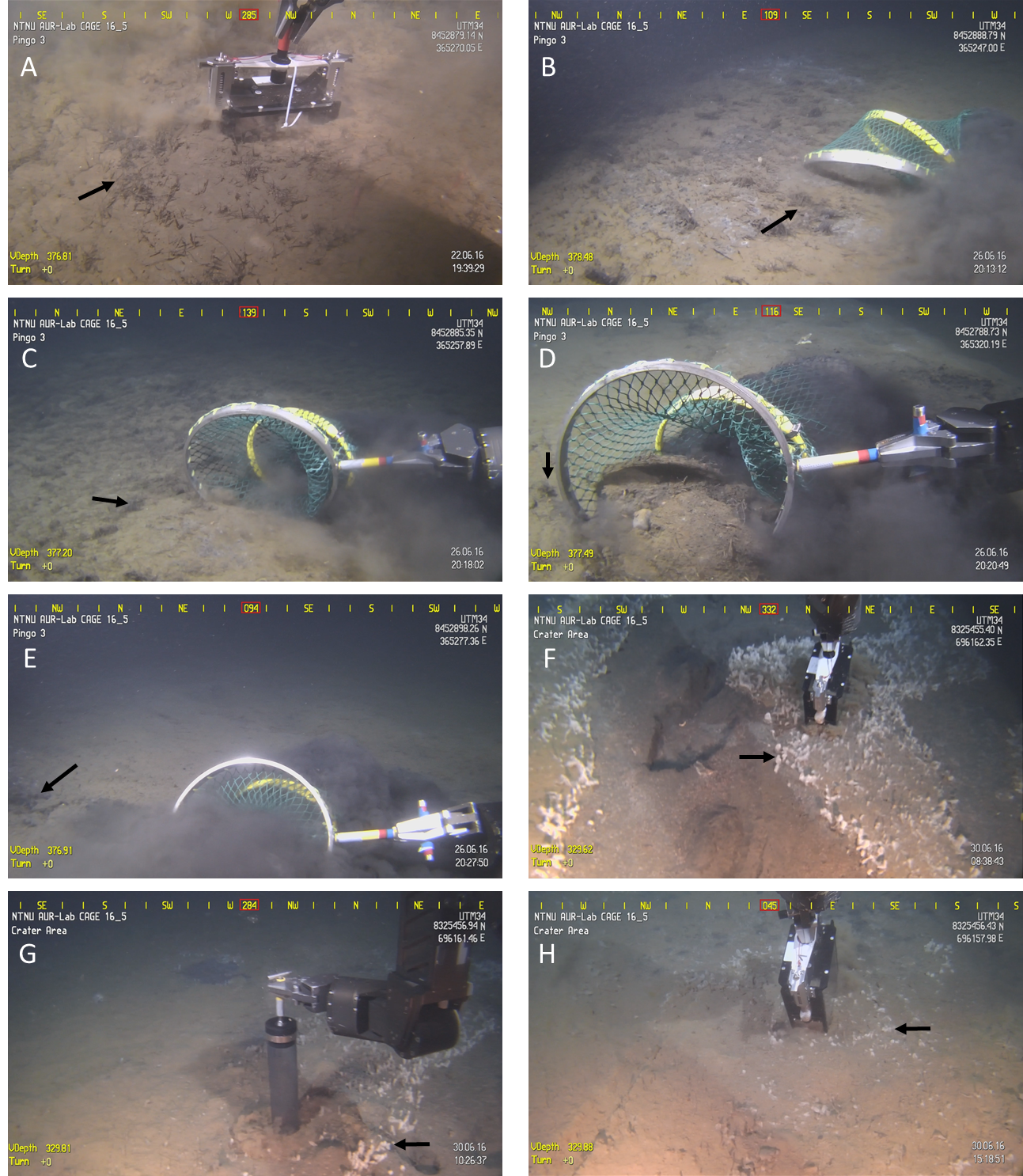

Supplement: S1 Fig — Visual overviews of the sampling locations at the pingo sites (A-E) and crater sites (F-H). Images are video stills from the ROV 30K’s high definition video system. A: sample 1029 (blade core), B: sample 1078 (first scoop), C: sample 1078 (second scoop), D: 1078 (third scoop), E: 1078 (fourth scoop), F: 1123 (blade core), G: 1124 (push core) and H: 1125 (blade core). Arrows are shown to highlight the presence of frenulates on the seafloor. No visuals for sample 1054 are available since this sample was taken with a Van Veen grab operated directly from the ship. (TIF) [file pone.0209273.s001.tif]

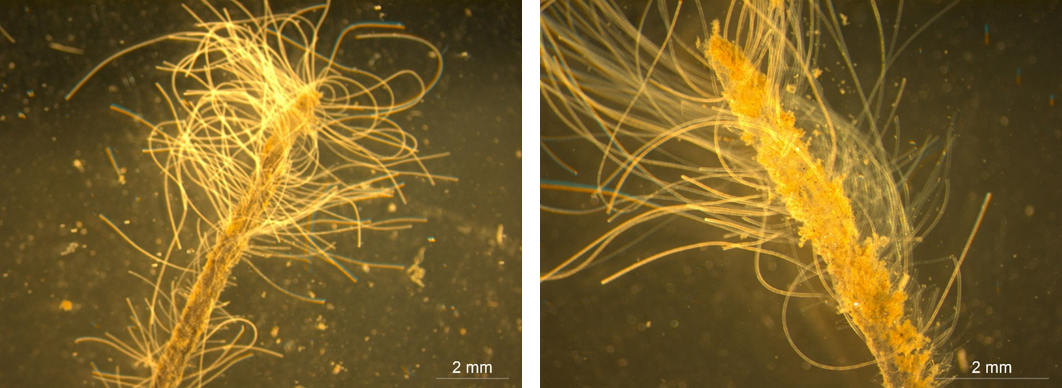

Supplement: S2 Fig — Close-up view of dense epibacterial colonies, seen on the anterior ends of the tubes of many individuals of the pingo and crater worms, giving a white, fuzzy appearance in videos and images. (TIF) [file pone.0209273.s002.tif]

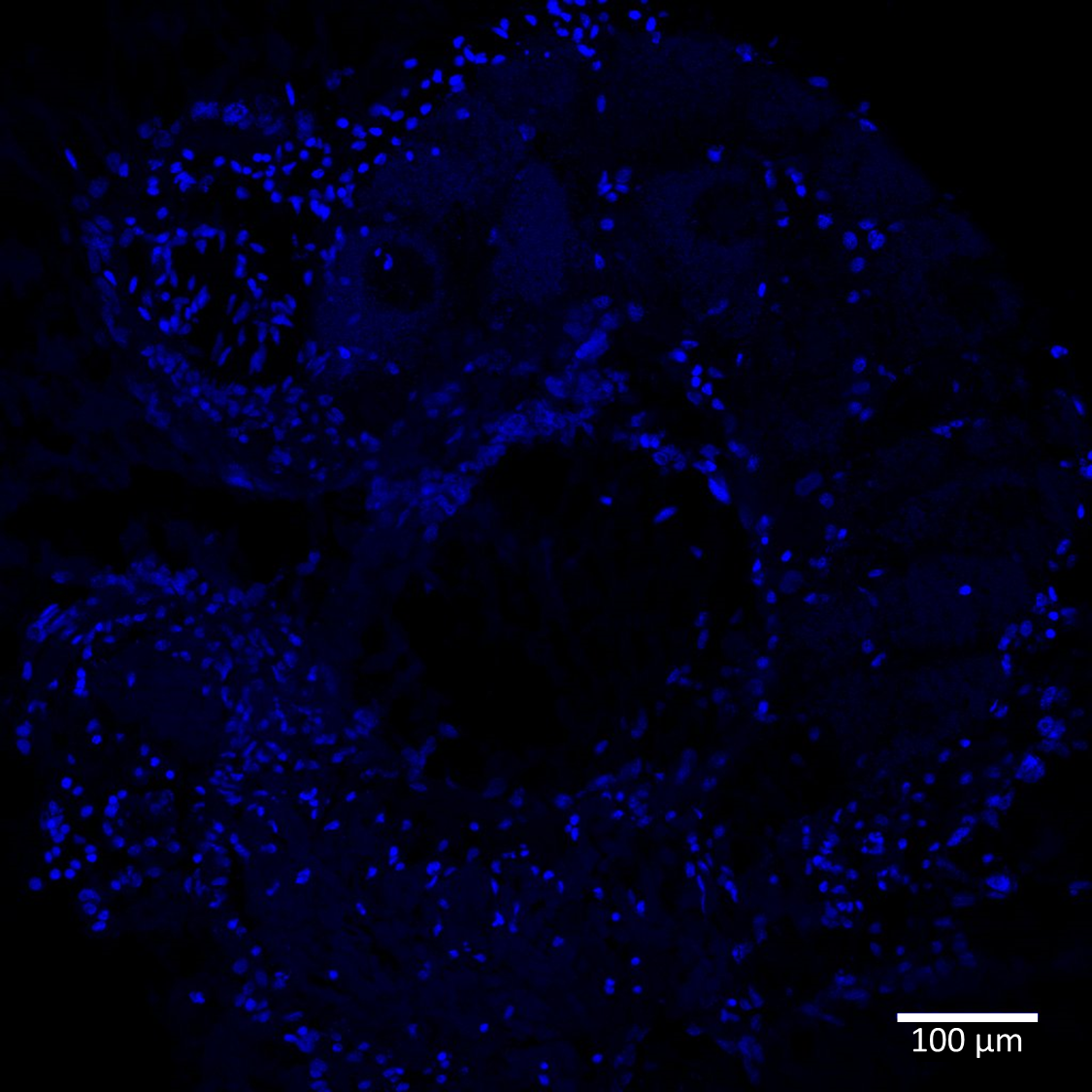

Supplement: S3 Fig — Epifluorescence micrograph of cross section of the trunk of a Spiochaetopterus worm from the crater site (sample 1124–6, negative control). Host/animal nuclei are stained with DAPI (blue). Hybridization did not occur and no fluorescence for bacterial symbionts were detected. (TIF) [file pone.0209273.s003.tif]

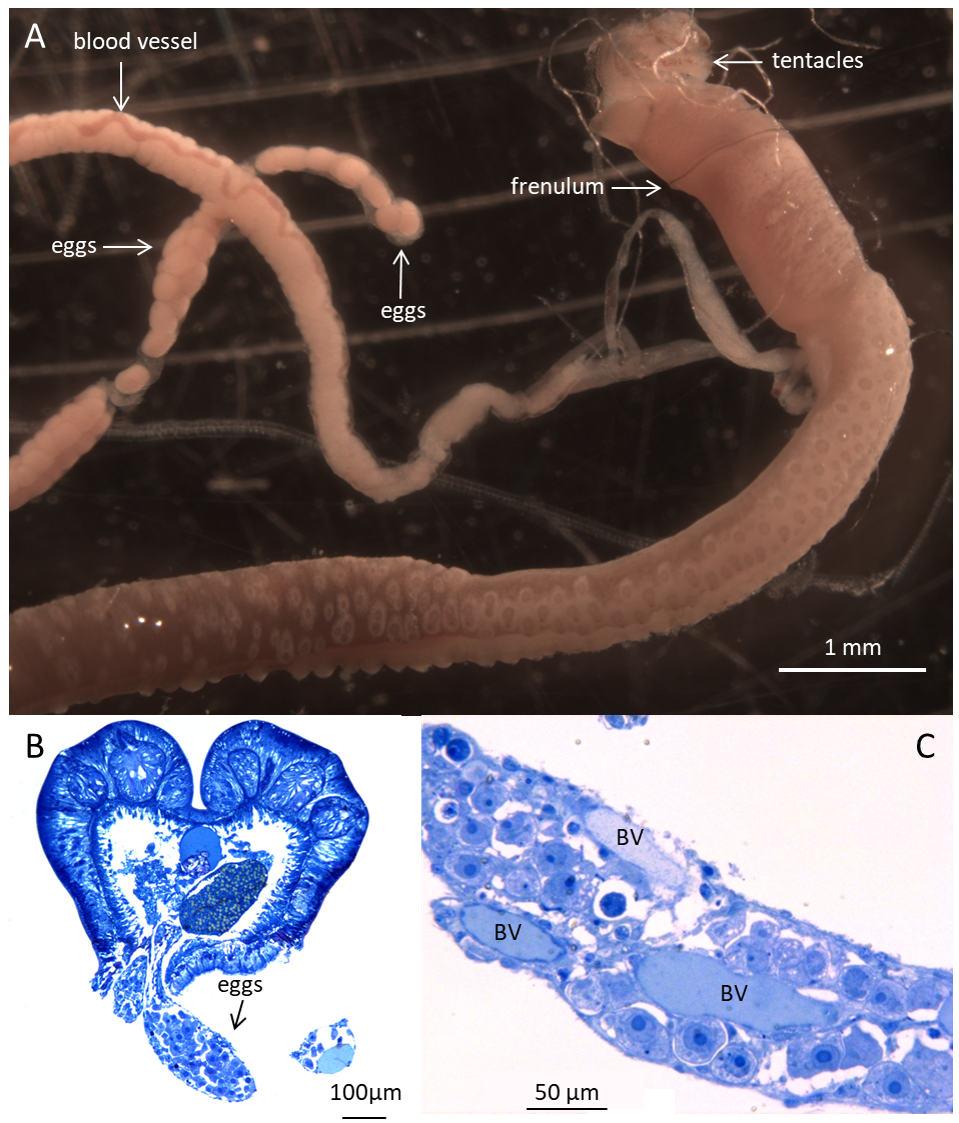

Supplement: S4 Fig — Oligobrachia clade individual with the ‘egg string’ feature. A: Stereozoom view of the two arms of the feature, beginning immediately posterior of the diaphragm on the ventral side of the animal. The string is lined by a continuous blood vessel on either side of which lie the eggs. B: Semithin cross section at the level of the diaphragm, clearly showing the outpocking of this egg string from the inner body with the broken epidermis. C- Close-up view of the egg string showing longitudinal sections of the blood vessels and their close vicinity to the oocytes. (TIF) [file pone.0209273.s004.tif]
