# Supplementary material for: Cryptic frenulates are the dominant chemosymbiotrophic fauna at Arctic and high latitude Atlantic cold seeps
Source: PLoS One. 2018 Dec 28;13(12):e0209273. doi: 10.1371/journal.pone.0209273 (PMC6310283; doi:10.1371/journal.pone.0209273)
Supplement: S1 Table — List of all the pingo and crater samples used in this study. Single individuals were often used for multiple analyses (different segments for different analyses), particularly if large or whole segments could be retrieved from the tubes. In addition to the pingo and crater samples, 13 worms from the Lofoten canyons, 1 from Nyegga, 1 from the Laptev Sea and 4 Sclerolinum samples from Storegga were additionally used for DNA analysis of the worms (mtCOI sequencing). (PDF) [file pone.0209273.s005.pdf]

Sample number	site and sampling location	morphological measurements	mtCOI sequencing	APS and pmoA sequencing	16S rRNA sequencing	SEM	TEM	FISH
1029 two unnumbered individuals	pingos (GHP3)	x						
1029-1	pingos (GHP3)	x						
1029-3	pingos (GHP3)	x						
1054-3	pingos (GHP3)	x				x	x	
1054-4	pingos (GHP3)	x						
1054-5	pingos (GHP3)	x	x					
1054-6	pingos (GHP3)		x	x	x			
1054-8	pingos (GHP3)							
1078-1	pingos (GHP3)	x			x	x	x	x
1078-2	pingos (GHP3)	x	x				x	
1078-3	pingos (GHP3)		x				x	
1078-4	pingos (GHP3)		x				x	
1078-5	pingos (GHP3)	x						
1078-6	pingos (GHP3)	x						
1078-7	pingos (GHP3)	x						
1078-8	pingos (GHP3)	x	x					
1078-9	pingos (GHP3)	x						x
1078-10	pingos (GHP3)					x	x	
1078-11	pingos (GHP3)	x						
1078-12	pingos (GHP3)	x						
1078-13	pingos (GHP3)	x		x	x	x	x	x
1078-14	pingos (GHP3)	x	x			x		
1078-15	pingos (GHP3)	x	x (didn't amplify)		x			
1078-20	pingos (GHP3)	x						
1029-3	pingos (GHP3)					x		
1054-6	pingos (GHP3)		x					
1125-1	craters (Yin Yang)		x					
1125-3	craters (Yin Yang)		x					

1125-6	craters (Yin Yang)				x		x
1125-7	craters (Yin Yang)			x	x		
1125-9	craters (Yin Yang)		x		x		
1123-1	craters (Yin Yang)	x	x (didn't amplify)				
1123-2	craters (Yin Yang)	x	x (didn't amplify)				
1123-3	craters (Yin Yang)	x					
1123-4	craters (Yin Yang)	x					
1123-5	craters (Yin Yang)	x					
1123-12	craters (Yin Yang)	x					
1123-13	craters (Yin Yang)	x					
1123-3	craters (Yin Yang)	x					
1123-5	craters (Yin Yang)		x		x		
1123-12	craters (Yin Yang)				x	x	
1124-1	craters (Yin Yang)	x	x	x			x
1124-2	craters (Yin Yang)	x	x	x			
1124-3	craters (Yin Yang)	x	x (didn't amplify)	x			
1124-4	craters (Yin Yang)					x	
1124-5	craters (Yin Yang)	x					
1124-6 (not a frenulate)	craters (Yin Yang)	x	x (didn't amplify)				x
1124-7	craters (Yin Yang)	x					
1124-8	craters (Yin Yang)	x					
1125-1	craters (Yin Yang)	x	x			x	
1125-3	craters (Yin Yang)		x				
1125-4	craters (Yin Yang)	x	x (didn't amplify)	x	x	x	
1125-5	craters (Yin Yang)	x		x	x	x	
1125-6	craters (Yin Yang)	x					x
1125-7	craters (Yin Yang)	x		x			
1125-8	craters (Yin Yang)	x					
1125-9	craters (Yin Yang)	x					
